# Glucose- but Not Rice-Based Oral Rehydration Therapy Enhances the Production of Virulence Determinants in the Human Pathogen *Vibrio cholerae*

**DOI:** 10.1371/journal.pntd.0003347

**Published:** 2014-12-04

**Authors:** Juliane Kühn, Flavio Finger, Enrico Bertuzzo, Sandrine Borgeaud, Marino Gatto, Andrea Rinaldo, Melanie Blokesch

**Affiliations:** 1 Laboratory of Molecular Microbiology, Global Health Institute, School of Life Sciences, Ecole Polytechnique Fédérale de Lausanne (EPFL), Lausanne, Switzerland; 2 Laboratory of Ecohydrology, Environmental Engineering Institute, School of Architecture, Civil and Environmental Engineering, Ecole Polytechnique Fédérale de Lausanne (EPFL), Lausanne, Switzerland; 3 Dipartimento di Elettronica Informazione & Bioingegneria, Politecnico di Milano, Milan, Italy; 4 Dipartimento ICEA, Universitá di Padova, Padova, Italy; University of Tennessee, United States of America

## Abstract

Despite major attempts to prevent cholera transmission, millions of people worldwide still must address this devastating disease. Cholera research has so far mainly focused on the causative agent, the bacterium *Vibrio cholerae*, or on disease treatment, but rarely were results from both fields interconnected. Indeed, the treatment of this severe diarrheal disease is mostly accomplished by oral rehydration therapy (ORT), whereby water and electrolytes are replenished. Commonly distributed oral rehydration salts also contain glucose. Here, we analyzed the effects of glucose and alternative carbon sources on the production of virulence determinants in the causative agent of cholera, the bacterium *Vibrio cholerae* during *in vitro* experimentation. We demonstrate that virulence gene expression and the production of cholera toxin are enhanced in the presence of glucose or similarly transported sugars in a ToxR-, TcpP- and ToxT-dependent manner. The virulence genes were significantly less expressed if alternative non-PTS carbon sources, including rice-based starch, were utilized. Notably, even though glucose-based ORT is commonly used, field studies indicated that rice-based ORT performs better. We therefore used a spatially explicit epidemiological model to demonstrate that the better performing rice-based ORT could have a significant impact on epidemic progression based on the recent outbreak of cholera in Haiti. Our results strongly support a change of carbon source for the treatment of cholera, especially in epidemic settings.

## Author Summary

Cholera research has so far mainly focused on the causative agent, the bacterium *Vibrio cholerae*, or on disease treatment, but rarely were results from both fields interconnected. Indeed, the treatment of this severe diarrheal disease is mostly accomplished by oral rehydration therapy (ORT). ORT aims at rehydrating patients through the provision of water and oral rehydration salts; the latter being composed of electrolytes as well as glucose as a carbon source. Although glucose-based ORS is commonly used to treat diarrheal diseases and is recommended by the WHO, field studies on cholera indicated that rice-based ORT performs better than glucose-based ORT. Here, we investigated the impact that glucose, starch, or other carbon sources exert on *V. cholerae*. We demonstrated that glucose leads to an increased expression of the major virulence genes in the pathogen and, accordingly, to an enhanced production of cholera toxin during *in vitro* experimentation. Because the cholera toxin is primarily responsible for the severe symptoms that are associated with the disease, our study highlights the negative effects of glucose-based ORT. Next, we used a spatially explicit epidemiological model to demonstrate that the better performing rice-based ORS could have a significant impact on epidemic progression based on the recent outbreak of cholera in Haiti.

## Introduction

The diarrheal disease cholera remains a major problem in developing countries. In 2012, almost 250'000 cases were reported to the WHO; however estimated numbers, including non-reported cases, are argued to reach several million cases every year. The recent disease outbreak in Haiti demonstrated the devastating effects of cholera epidemics. Because of these dramatic consequences, the problem of how to stop an epidemic at an early stage, or at least to slow it down, was addressed and the employment of general intervention strategies was discussed. In this context, mathematical models were developed and used to predict the outcome of major interventions, such as vaccination or extended use of antibiotics and sanitation [1]–[6]. Remarkably, more general treatment strategies have not been considered. This fact appears surprising because, without treatment, the case fatality rate for severe cholera is approximately 50%; however, if handled properly, nearly all deaths can be avoided. The general treatment of cholera patients is based on a so-called oral rehydration therapy (ORT), which is a cost-effective and easily applicable method to replace lost fluids and electrolytes. For the latter purpose, the administered solution contains a mixture of several compounds that were designated oral rehydration salts (ORS), including sodium, chloride, and potassium ions as well as glucose. Indeed, glucose is the most commonly added carbohydrate because it stimulates sodium and therefore, water absorption in the small intestine [7]. However, in field studies, it was shown that ORT might be improved by the substitution of the carbon source (or through the addition of amino acids and supplementation with trace elements) [8]. The molecular mechanisms underlying these findings remain to be resolved. Moreover, a meta-analysis comparing the treatment with standard, glucose-based versus rice-based ORS illustrated the beneficial effects of the latter composition, such as reduced episodes of vomiting, a decrease of the stool volume, and a shortened recovery time [9]–[12].

**Figure 1:**
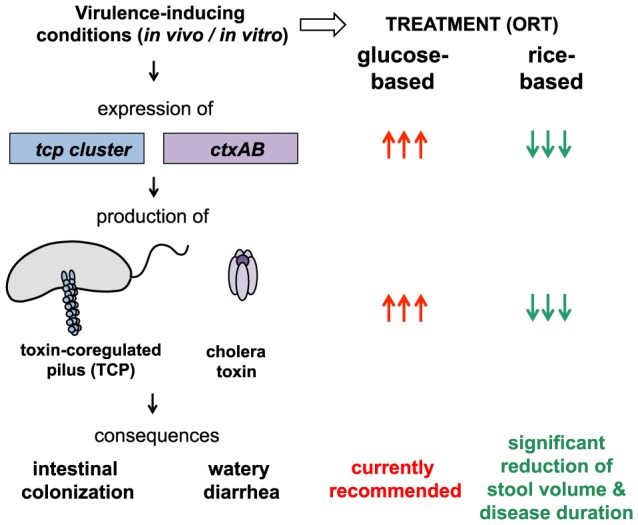
Glucose-based oral rehydration therapy (ORT) might enhance the production of virulence determinates in *V. cholerae* thereby extending the duration of the disease and worsen the symptoms compared to rice-based ORT. Upon virulence-inducing conditions (e.g., in the human intestine or by *in vitro* provision of bicarbonate as inducer [21], [29]) the human pathogen *V. cholerae* expresses amongst others the virulence genes of the *tcp* and *ctx* clusters, which ultimately leads to the synthesis and assembly of the toxin-coregulated pilus (TCP) and the production of cholera toxin, respectively. The TCP forms a type IV pilus structure on the bacterial surface, which is required for intestinal colonization. The cholera toxin indirectly leads to efflux of ions (concomitantly with water), which causes the dramatic watery diarrhea, the hallmark of cholera. In this study, we assessed the expression of the *tcp* and *ctx* genes and the production of cholera toxin under virulence-inducing conditions and in the presence of diverse carbon sources. Our data indicate that glucose strongly enhances the virulence potential of the pathogen (indicated by the arrows). Thus, based on the better performance of rice-based ORT as described in field studies and on our model-guided prediction of cholera transmission under epidemic settings we suggest that rice-based ORT should be reconsidered for the treatment of cholera.

The aim of this study was to unravel the molecular mechanisms underlying the better performance of rice-based treatments (Fig. 1). To do so, we analyzed the virulence gene expression of the causative agent of cholera, specifically the bacterium *Vibrio cholerae*, when cultured under conditions resembling ORT. Our results show that, in the presence of glucose, virulence-associated genes were induced and cholera toxin was produced and this *in vitro* induction was dependent on the main virulence regulatory proteins ToxR, TcpP and ToxT [13]. However, substituting glucose in ORT by an alternative non-PTS carbon source, such as rice-derived starch, led to the significantly reduced expression of virulence determinants in *V. cholerae*, which would have beneficial effects on disease progression. Based on this evidence, the impact of such optimized treatment on the outcome of cholera epidemics was simulated using a mathematical model, which recapitulates the recent Haitian cholera outbreak [4]. Our results indicate that rice-based treatment could have significantly reduced the burden imposed on the Haitian population.

## Materials and Methods

### Bacterial strains, media and growth conditions

The *Vibrio cholerae* strains that were used in this study are El Tor Inaba strain A1552 [14], El Tor Inaba strain N16961 [15], classical Ogawa strain O395 (India, 1964) [16], and derivatives of those strains (see Supporting Table S1). All strains belong to serogroup O1. *V. cholerae* strains were grown as overnight cultures in Luria broth (LB) or M9 minimal medium at 30°C under shaking conditions. Antibiotics were added at the following concentrations when required: kanamycin at 75 µg/ml or ampicillin at 100 µg/ml.

### Construction of *V. cholerae* genetically-modified strains

The genes *toxR*, *ptsG*, and *nagE* were deleted from the parental *V. cholerae* strain (A1552) using the TransFLP gene disruption method [17]–[19], which is based on natural transformation and FLP recombination. The same method was applied to introduce a functional *hapR* gene into strain N16961 to acquire strain N16961rep (the natural transformation was performed in the presence of a *hapR*-complementing plasmid). To delete the genes *tcpP* and *toxT* from the parental strain A1552, a gene-disruption method based on the counter-selectable plasmid pGP704-Sac28 [20] was used. The oligonucleotide sequences that were used to construct the transforming PCR fragments or plasmids pGP704-28-SacB-ΔVC0826 and pGP704Sac28ΔtoxT-II are indicated in Supporting Table S2. Genomic DNA from *V. cholerae* strain A1552 served as a template.

### *In vitro* induction of virulence gene expression

A detailed protocol for virulence gene expression in *V. cholerae* is provided in the Supporting Information (Supporting Text S1). Briefly, bacteria were grown in a M9 minimal salt medium (Sigma-Aldrich, Buchs, Switzerland), which contained vitamins and casamino acids, as well as the carbon source of interest. Virulence gene expression was induced by the addition of sodium bicarbonate (Fluka, St. Louis, USA) as published for rich medium conditions [21]. Gene expression was evaluated using quantitative reverse transcription-based PCR (qRT-PCR) as described [22]. The gene-specific primers that were used for qRT-PCR are indicated in Supporting Table S2.

### Enzyme-linked immunosorbent assay (ELISA)

Cholera toxin (CT) concentrations were determined using a CT-ELISA, which was performed essentially as reported [23], with the exception that phosphate-citrate buffer containing sodium perborate was used for detection.

### Mathematical model of the cholera epidemic in Haiti

A mathematical model was applied to recapitulate the first year of the cholera outbreak that recently occurred in Haiti and to estimate the potential impact of rice-based ORT. In addition to the epidemiological processes that are relevant to cholera transmission, the model also takes hydrological pathogen transport, human mobility, and precipitation into account (see Supporting Text S1). Model parameters were estimated based on the available literature or calibrated using a Bayesian approach. The modeled progression of the cholera epidemic corresponded well with the observed cases (details on the spatial match of reported cases and simulation results are provided elsewhere [4], [6], [24], [25]; see also Supporting Text S1). Next, the described effects of the rice-based treatment were implemented. This is compared with standard glucose-based ORS by assuming that (a) during the actual course of the Haiti epidemic all symptomatic infected people received a glucose-based ORT, and (b) all parameters (namely patterns of human mobility, demography, bacterial ecology, and exposure rates) are assumed unaltered by the ORT except for the shedding rate and disease duration (according to [9]–[12]). Moreover, a 30-days lag period between the initial cholera case and the switch to the rice-based ORT was included because this timeframe seems reasonable to first confirm the causative agent of the diarrheal disease outbreak as being *V. cholerae* at the onset of the epidemic. Details regarding the model structure, the parameterization, and the validation can be found in the Supporting Information, which contains Supporting Text S1, Supporting Figures (Fig. S3, Fig. S4, Fig. S5, Fig. S6, and Fig. S7) and Supporting Tables (Table S4 and Table S5).

## Results

### Induction of virulence gene expression in *V. cholerae* in the presence and absence of glucose

This study aimed to investigate how different carbon sources that are used for cholera treatment influence the production of the major virulence determinants in *V. cholerae*, cholera toxin (CT) and the toxin-coregulated pilus (TCP)(Fig. 1). CT is an AB_5_-toxin, which consists of two different subunits that are encoded by the genes *ctxA* and *ctxB*. Whereas the B subunit is important for the binding to host cells, the A subunit indirectly activates the enzyme adenylate cyclase within gut epithelial cells, which leads to an increased secretion of chloride and concomitantly increased secretion of water (Fig. 1). CT alone can cause diarrhea, and the amount of CT intake was shown to be proportional to the severity of symptoms [26], [27]. The second major virulence factor of pathogenic *V. cholerae* strains, TCP, is important for intestinal colonization [28]. The pilus structure primarily consists of the major pilin TcpA, whereas the residual *tcp* gene products are important for the pilus structure and for its assembly (Fig. 1).

**Figure 2:**
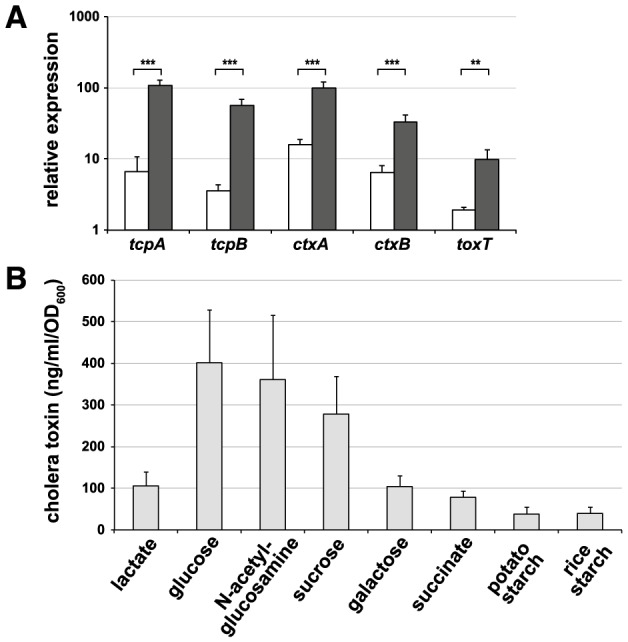
Expression and production of virulence factors in *V. cholerae* is enhanced in the presence of glucose. (**A**) Relative expression of virulence-associated genes in the *V. cholerae* wild-type strain A1552. The virulence inducer bicarbonate was present in all samples. Supplemented carbon sources were lactate and glucose as indicated by the white and black columns, respectively. Statistics were applied using Student's t-test (**, *P*≤0.01; ***, *P*≤0.001). (**B**) Quantification of cholera toxin that was produced upon growth on diverse carbon sources (as indicated). The data were normalized by the OD_600_ values of the bacterial culture. For rice starch-grown cells the OD_600_ value was deduced from the number of counted bacteria. All data represent averages from three independent biological experiments ± SD.

We first established an assay to monitor the expression of these major virulence genes when the bacteria were grown under conditions that somewhat mimic those conditions that are encountered in patients undergoing ORT. To this extent, we used a bicarbonate-mediated *in vitro* virulence induction protocol [21], [29] but changed the culture medium to minimal salts (resembling ORS) instead of the previously described rich-medium conditions. Using quantitative qRT-PCR, we demonstrated that, upon growth of the *V. cholerae* wild-type O1 El Tor strain (A1552) in the presence of glucose, the genes *ctxA, ctxB, tcpA* and *tcpB* were significantly induced whereas significantly lower induction was observable when lactate was used as alternative carbon source (Fig. 2A) or under virulence-non-inducing conditions (Fig. S1 and Table S3). To confirm that the reduced virulence gene expression is also reflected in reduced protein levels of the virulence determinants, we analyzed the accumulation of CT in culture supernatants using an ELISA. Indeed, the amount of cholera toxin decreased to ∼25% for cultures that were grown in the presence of lactate compared with glucose (Fig. 2B).

Because it has become evident in recent years that the regulation of virulence genes differs between diverse pathogenic isolates [30], [31], we applied our method to two other epidemic *V. cholerae* strains. N16961 is an El Tor O1 strain (as is strain A1552), whereas O395 is a classical O1 biotype strain [15], [16]. Indeed, these two strains also exhibited a glucose-dependent induction of virulence genes (Fig. S1), which indicates the general impact of carbon sources on the regulatory network of virulence-associated genes in *V. cholerae*.

### Effect of diverse carbon sources on virulence gene expression

Most bacteria transport preferred sugars, such as glucose, by so-called phosphoenolpyruvate-phosphotransferase systems (PTS); therefore, the corresponding carbohydrates are often denoted as PTS sugars. During the sugar uptake via the PTS, a phosphoryl group is transferred onto the imported sugar, which prepares the sugar for central metabolism. In contrast, the enzyme adenylate cyclase, which is responsible for the production of the secondary messenger cyclic AMP (cAMP), is activated when cells run short of PTS sugars. cAMP that is bound to the cAMP-receptor protein (CRP) is a crucial signaling compound and modulates several processes in bacteria [32]. Thus, we asked the question whether the observed virulence induction was specific to glucose, specific to PTS sugars, or a broader effect that was exerted by many carbon sources. Therefore, we tested other PTS sugars in addition to glucose (e.g., N-acetylglucosamine and sucrose) and observed a comparable induction of the virulence genes, as demonstrated for glucose (Tables 1 and 2). In contrast, galactose and succinate, which are two non-PTS carbon sources, led to highly reduced virulence expression (Table 1). More importantly, we demonstrated that *V. cholerae* induces the virulence determinants at significantly lower levels when starch was provided as the sole carbon source, which mimics the conditions of rice-based ORT (Table 1). This reduced virulence gene expression was again reflected in the accumulation of CT, which decreased to 10–25% for cultures that were grown in the presence of non-PTS carbon sources (e.g., lactate, galactose, succinate or starch) compared with glucose (Fig. 2B).

**Table 1:** Transcript levels of virulence factor encoding genes in the presence of different carbon sources.

gene	glc/lactate	glc/galactose	glc/succinate	glc/GlcNAc	glc/sucrose	glc/potato starch	glc/rice starch	glc/75 mM glc	glc/111 mM glc
*tcpA*	16.7	9.6	32.1	0.9	1.1	2.7	4.7	1.1	1.4
*tcpB*	15.7	10.3	18.9	1.0	1.2	3.4	8.9	1.1	1.5
*ctxA*	6.2	6.3	5.0	1.0	1.1	2.5	3.5	1.2	1.7
*ctxB*	5.1	5.7	4.1	0.9	1.0	2.5	3.5	1.4	1.8

Virulence gene expression in *V. cholerae* cultures that were grown in the presence of 50 mM glucose (glc) compared with cells that were grown with the indicated carbon sources. The fold differences are indicated. GlcNAc, N-acetylglucosamine.

**Table 2:** PTS sugar-dependent virulence gene expression requires sugar uptake.

tested gene	WT (−)	WT (+)	Δpts (−)	Δpts (+)	fold difference WT (+)/Δpts (+)	statistics
**glucose**						
*tcpA*	16.9 (±1.5)	114.8 (±6.6)	14.5 (±7.7)	17.7 (±6.8)	6.5	***
*tcpB*	7.1 (±1.2)	59.9 (±8.0)	6.6 (±1.9)	9.2 (±3.6)	6.5	**
*ctxA*	21.5 (±3.2)	83.3 (±18.2)	19.8 (±7.6)	20.2 (±9.1)	4.1	*
*ctxB*	7.5 (±0.6)	28.7 (±3.6)	5.9 (±2.2)	6.4 (±2.5)	4.5	**
*toxT*	5.0 (±1.2)	11.5 (±3.4)	2.9 (±0.3)	2.2 (±0.5)	5.3	*
**GlcNAc**						
*tcpA*	12.8 (±2.0)	123.5 (±17.2)	8.4 (±2.9)	3.9 (±2.9)	31.3	**
*tcpB*	6.5 (±1.4)	65.6 (±7.9)	3.9 (±0.7)	2.5 (±0.7)	26.0	**
*ctxA*	18.9 (±7.0)	99.9 (±23.5)	17.4 (±5.9)	14.6 (±5.3)	6.8	*
*ctxB*	6.8 (±2.3)	30.6 (±4.7)	5.6 (±1.6)	4.9 (±2.0)	6.2	**
*toxT*	4.5 (±0.9)	9.6 (±3.9)	2.3 (±0.6)	1.6 (±0.1)	6.1	*ns*
**sucrose**						
*tcpA*	11.5 (±2.5)	91.9 (±17.1)	2.4 (±0.8)	3.3 (±1.7)	27.4	*
*tcpB*	5.8 (±0.4)	49.2 (±7.3)	2.2 (±0.2)	2.8 (±0.4)	17.7	**
*ctxA*	15.4 (±5.6)	69.0 (±11.0)	8.1 (±1.9)	12.4 (±7.1)	5.6	**
*ctxB*	5.0 (±1.7)	22.4 (±2.1)	2.4 (±0.6)	4.2 (±2.8)	5.3	**
*toxT*	3.7 (±0.7)	8.4 (±4.4)	1.4 (±0.1)	2.1 (±0.9)	4.1	*ns*

Bacterial cultures contained the indicated carbon sources (glucose, GlcNAc, sucrose) plus lactate to maintain growth. Strains tested for the expression of the indicated genes (relative to the housekeeping gene *gyrA*) were: wild-type (WT), and the respective PTS mutants for glucose (ΔptsG), GlcNAc (ΔnagE), and sucrose (ΔscrA) in the absence (−) or presence (+) of bicarbonate as virulence inducer. Average of at least three independent experiments (± SD) are depicted. Statistics were applied using Student's t-test comparing the WT (+) and Δpts (+) conditions (* *P*≤0.05; ** *P*≤0.01; *** *P*≤0.001; *ns*, not statistically different).

### Sugar transport is crucial for the induction of the virulence genes

To elucidate whether the sugar-dependent virulence induction is linked to the transport of the PTS sugars we employed genetically modified *V. cholerae* strains, which lacked the PTS transporters for glucose, N-acetylglucosamine, or sucrose. In these strains, the signaling pathway that was described above is disrupted, thereby allowing cAMP to accumulate in the bacterial cell. As shown in Table 2, the increase of virulence gene expression upon provision of the inducer bicarbonate was abolished in those strains, which were cultivated in the presence of the corresponding PTS sugars (with the concomitant addition of lactate to maintain growth). Therefore, we conclude that the expression of the virulence genes is not dependent on any particular sugar *per se* but is dependent on the PTS-coupled regulatory circuit.

### Role of quorum sensing and the regulatory protein HapR

The PTS-dependent signaling pathway (including the secondary messenger cAMP) is part of a process known as carbon catabolite repression in bacteria [32]. This mechanism allows bacteria to first utilize preferred sugars through the repression of uptake complexes and catabolic enzymes that are required for the utilization of less-preferred carbon sources. Earlier studies have indicated that carbon catabolite repression also acts as a modulator of quorum sensing (QS) in *V. cholerae* [33]. QS is commonly used by bacteria to assess population densities and to respond appropriately to these [34]. The master regulator of QS in *V. cholerae* is the protein HapR, which is produced at high cell density and which represses the virulence genes [35], [36]. Consistent with these earlier studies, we observed increased virulence gene expression in a *hapR* mutant strain when compared with the wild-type strain (Fig. S2). Furthermore, the *V. cholerae* strain N16961 [15], which has a frameshift mutation in *hapR* that renders the HapR protein non-functional [35], also showed increased virulence gene expression compared with a genetically modified variant of N16961 in which the *hapR* gene was repaired (Fig. S2). However, the *hapR* mutant strain still displayed glucose-responsiveness with respect to virulence gene expression, which indicated that carbon catabolite repression also influences virulence in a QS-independent manner (Fig. S2).

### *In vitro* virulence-inducing conditions reflect the *in vivo* situation

**Figure 3:**
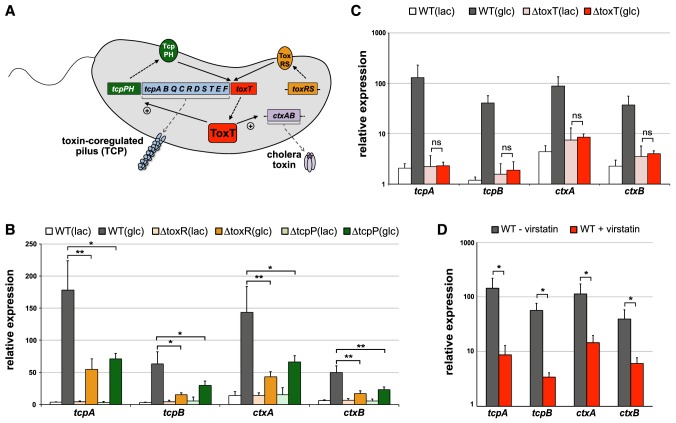
Expression of virulence determinants is dependent on the common virulence regulators under *in vitro* conditions. (**A**) Simplified overview of the regulatory cascade that is involved in virulence gene expression in *V. cholerae*. *toxT* expression occurs upon activation by the regulatory proteins ToxR and TcpP (complexed with ToxS and TcpH, respectively) as indicated by the arrows (solid lines). Next, the synthesized ToxT protein (dashed arrow) induces the expression of the *tcp* cluster and the *ctxAB* operon, which ultimately leads to the production of TCP and CT. Elements acting upstream *tcpPH* and *toxRS* are excluded for sake of simplicity. Scheme is based on [13]. (**B+C**) Relative expression of the genes *tcpA, tcpB, ctxA*, and *ctxB* in diverse *V. cholerae* strains, which were grown under virulence-inducing conditions in the presence of lactate (lac) or glucose (glc). Strains tested as indicated above the graphs: panel B: wild type *V. cholerae* strain (WT; A1552-Tn*tfoX*) and its *toxR* and *tcpP*-negative derivatives (ΔtoxR and ΔtcpP). panel C: wild type (WT; A1552) and ΔtoxT (A1552ΔtoxT). (**D**) As in panels B and C except that the *V. cholerae* wild-type strain was grown under glucose-containing virulence-inducing conditions in the presence of DMSO (gray bars) or virstatin (50 µM dissolved in DMSO; red bars). The values shown in panels B, C, and D represent averages of at least three independent biological replicates (± SD) Statistics were applied on pairwise comparisons using Student's t-test (ns, non-significant; *, *P*≤0.05; **, *P*≤0.01; ***, *P*≤0.001).

As the aforementioned observations were based on *in vitro* data the question arose as to how these data compare to the situation within human patients. It should be noted that field studies indicated that rice-based (e.g., starch-based) ORT performs better compared to standard glucose-based ORT though the underlying mechanism remained elusive [9]–[12]. Apart from these important field study data it is difficult or even impossible to test the *in vivo* situation under laboratory conditions (e.g., outside the human intestine). The reason for that is that animal models do not recapitulate the disease well. Indeed, both adult rabbits and adult mice are naturally resistant to infection with the pathogen. Thus, the most prominent animal models of cholera are the rabbit ileal loop model and the infant mice model (for recent review see [37]). The first model primarily reflects the enterotoxicity of the bacteria measured by the accumulation of fluid within ligated intestinal loops. However, this model does not take the natural oral infection route into consideration (e.g., external cues encountered throughout the gastrointestinal passage). Moreover, due to the feature of the model as being a closed system it does not allow any ORT treatment studies. Lately, infant mice (suckling mice) have been widely used to study virulence factors of *V. cholerae*. The infant mice model allows elucidating the potential of the pathogen to grow inside the animal and to colonize the small intestine. However, infant mice do not develop severe diarrhea and fluid replacement by ORT is therefore impossible. Notably, these models have been extremely valuable for deciphering the pathogenicity of the bacterial pathogen [37]. For example the involvement of TCP in intestinal colonization was established based on the suckling mice model [28](Fig. 1), which was later confirmed to also be relevant in humans based on volunteer studies [38]. We therefore reasoned that the relevance of our *in vitro* data would be significantly strengthened if we could confirm that the observed *in vitro* virulence expression would still be subject to the well-established regulatory cascade of virulence induction in *V. cholerae* (“the ToxR regulon” [39]), which includes the main regulatory proteins TcpP, ToxR [28], [40], and ToxT [41] (for review see [13], [42])(Fig. 3A).

To this extent, we first generated *V. cholerae* knockout strains lacking either *toxR* or *tcpP* (Fig. 3A; Supporting Table 1) and subjected these strains and the parental wild-type strain to *in vitro* virulence induction in the presence of different carbon sources. Importantly, we saw a strong and statistically significant decrease in the expression of all tested virulence genes (*tcpA, tcpB, ctxA, ctxB*) in the presence of glucose when TcpP and ToxR were absent (Fig. 3B). As both of these regulatory proteins are required for the production of ToxT (Fig. 3A), the master regulator of virulence, which directly activates the *tcp* and *ctx* gene cluster by binding to so called toxboxes [13], we next tested the contribution of ToxT to virulence induction under the here described *in vitro* virulence induction conditions. Indeed, the expression of *toxT* under virulence-inducing conditions was significantly increased in the presence of glucose compared to lactate (Fig. 2A) and this increase was dependent on the presence of the respective PTS transport system (Table 2). Importantly, when we deleted the *toxT* gene or grew the wild-type cells in the presence of the ToxT inhibitor virstatin [43], [44] the glucose-dependent virulence induction was absent or significantly reduced (Figs. 3C and 3D) highlighting the importance of ToxT for *in vitro* virulence induction. Thus, as the *in vitro* induction of virulence determinants was dependent on the main regulatory proteins TcpP, ToxR, and ToxT we concluded that our approach recapitulates the *in vivo* situation to a certain extent and that the here described glucose-dependent expression of the main pathogenicity genes most likely also occurs in human patients. This finding would be consistent with the increased disease burden associated with glucose-based ORT compared to rice-based ORT [9]–[12].

### Starch as an alternative carbon source in ORT

**Figure 4:**
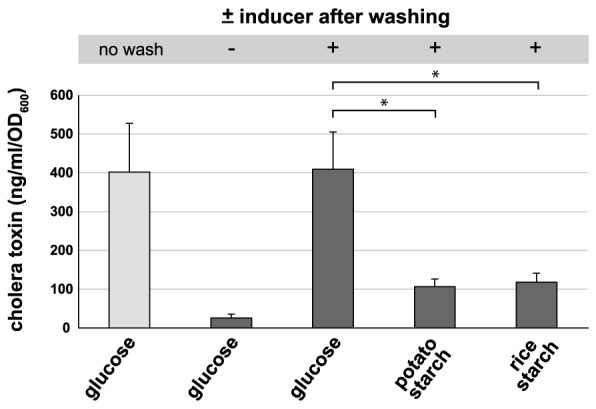
Shifting *V. cholerae* from glucose-based to starch-based conditions leads to a decrease in CT production. Bacterial cultures were induced for virulence in the presence of glucose for 30 min, washed (where indicated) and shifted to virulence-non-inducing or virulence-inducing medium containing either glucose or starch. The amount of CT was determined as described for Figure 2. Statistically significant differences were determined through Student's t-test (* *P*≤0.05).

Because we demonstrated above that starch does not support virulence gene expression to the same high level as glucose does, we aimed to test whether virulence could still be lowered by starch if previously induced. Indeed, such a scenario would occur at the onset of cholera symptoms and upon initial treatment with glucose-based ORS. Thus, we grew *V. cholerae* in glucose-containing virulence-inducing medium for a short period before washing the bacteria and shifting them to fresh medium, which contained either glucose or starch. Using this experimental approach, we observed a significantly reduced amount of CT (down to ∼25%) in starch-shifted cultures compared with the glucose-grown bacteria (Fig. 4). These data suggest that the administration of rice-based ORS, even after the initial virulence induction, would still significantly reduce CT accumulation along with reduced cholera symptoms.

### Modeling the impact of rice-based ORS on the Haitian cholera epidemic

**Figure 5:**
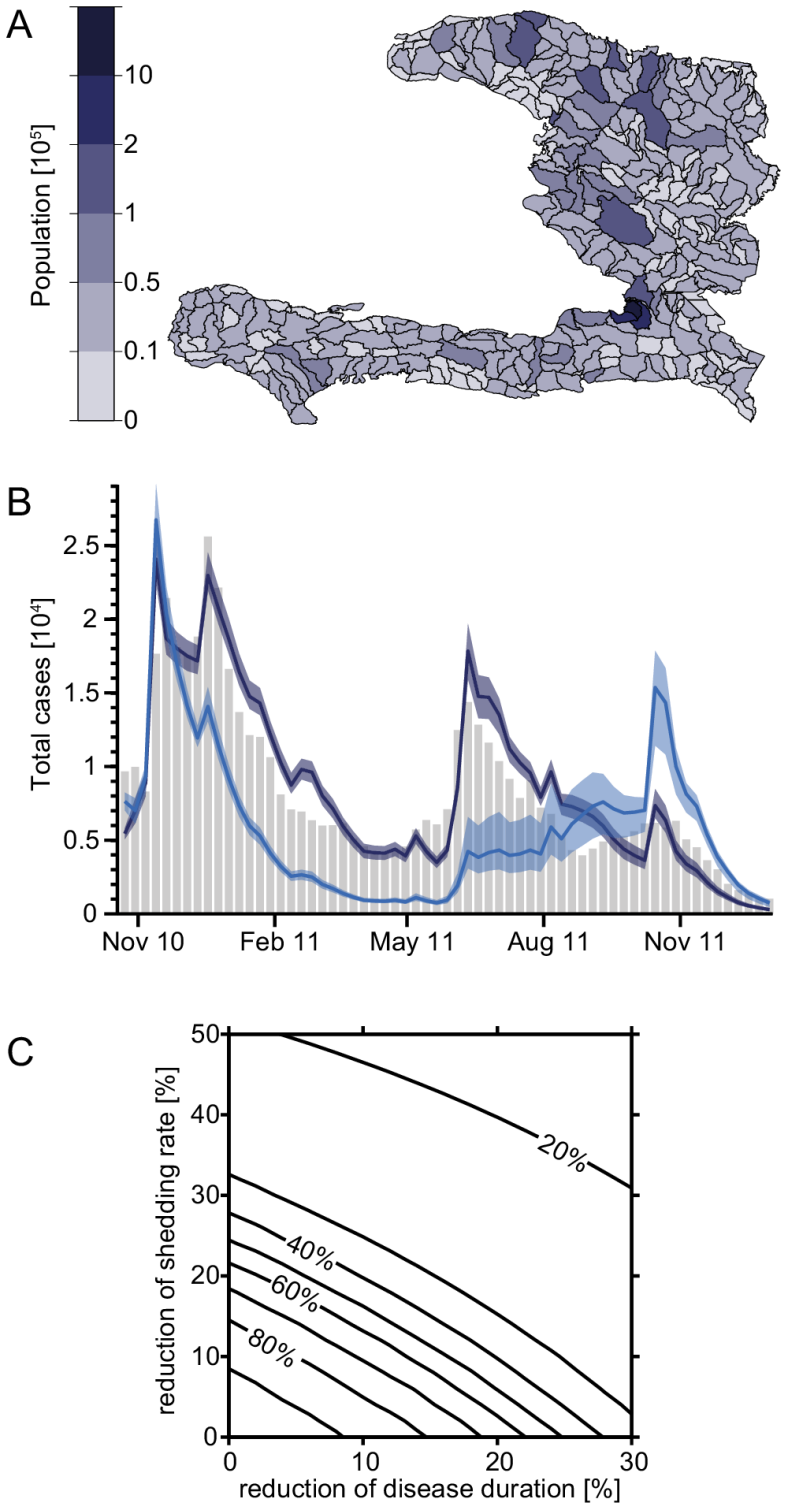
Modeling the effect of rice-based ORT on the Haitian cholera outbreak. (**A**) Computational subunits of Haiti with population sizes. Each unit corresponds to one node in our model. (**B**) Evolution of the epidemic over time for the entire country. Observed cases (grey bars), calibrated model (dark blue), and model runs with a ten percent reduction of symptomatic shedding rate as well as disease duration due to rice-based ORT (light blue). Shaded areas show the 95% confidence intervals resulting from parameter uncertainty; the solid lines show the median trajectory. The replacement of glucose-based with rice-based ORT was assumed to take place 30 days after the onset of the epidemic. (**C**) Evolution of the epidemics based on shedding rate and disease duration. Contour plot of the percentage of total cases in the whole country until December 2011, predicted by the model when applying variable reductions of the shedding rate (y-axis) and disease duration (x-axis), compared to the cases predicted by the calibrated model.

The data that were presented above provide a molecular explanation for why rice-based ORS performed better in field studies [9]–[12]. However, one wonders whether disease shortenings and reduced stool output, as described for rice-based ORT, would have any impact on large-scale patterns of cholera epidemics. We therefore used a spatially explicit epidemiological model [1], [4], [6] to recapitulate the recent Haitian cholera outbreak. The model consists of 365 nodes (human communities) that are spread over the Haitian territory (Fig. 5A).

Quantifying the reduction of shedding rate and disease duration is not easy. Our *in vitro* experiments suggest a strong reduction of the amount of CT, which matches the observed reduction of shedding rates from field studies [9]–[12]. This reduction can be as high as 50% (especially compared to the glucose-based ORS, which was recommended by the WHO before 2002 (WHO-ORS); see discussion). Reduction of diarrhea duration is smaller and usually no larger than 30%. If one assumes a 10% reduction in both duration and shedding rate for rice-based compared with glucose-based ORT (for HYPO-ORS; [12]), the model predicts a considerable decrease in disease incidence over the entire country (Fig. 5B). Indeed the total number of cholera cases within the first 14 months of the epidemic would be reduced from 520,000 cases (as reported by the Haitian Ministry of Health and available online at http://mspp.gouv.ht; our model predicts 535,000 cases) to 375,000 cases (i.e. 30% [22%–39%] less total cases until the end of 2011) according to the model. More importantly, if these parameters could be reduced by 15%, then the total number of cholera cases would drop by 59% [47%–67%], and if the parameters were reduced by 20%, the number of cases would even decrease by 74% [71%–76%] (Fig. 5C). The ranges of variation shown reflect the 2.5^th^–97.5^th^ percentiles of the uncertainty related to parameter estimation. Such behavior (i.e. the more than doubled reduction of total infections owing to a 10% to 20% reduction in bacterial shedding and disease duration) is typical of nonlinear epidemiological models [6]. Interestingly, owing to a higher number of susceptibles (Fig. S7 and Supporting Text S1), a larger number of cholera cases would have been predicted for November 2011, one year after the initial onset of the outbreak, most probably triggered by important rainfall events, which have been shown to play a major role in the dynamics of the epidemic [4], [45]. However, such a one-year time span would have allowed other intervention strategies to be put into place, which could potentially avoid later cholera case peaks e.g., by reducing exposure rates via improved water sanitation.

## Discussion

Cholera remains a major social emergency in developing countries and despite major research efforts this disease is far from being eradicated. Here, we aimed at understanding why an alternative cholera treatment, which relies on rice-based instead of glucose-based ORS, has beneficial effects on disease outcome as reported in field studies (Fig. 1). For this purpose, we tested the expression of virulence-associated genes in *V. cholerae* using defined virulence-inducing minimal medium conditions and a variety of carbon sources. Using this approach, we demonstrated that the virulence genes were upregulated in different clinical isolates of *V. cholerae* as long as those strains were grown in the presence of glucose or other PTS sugars (Fig. 2A, Fig. S1 and Table 1). The amount of cholera toxin produced under these conditions reflected the expression data (Fig. 2B). Because the cholera toxin is primarily responsible for the severe symptoms that are associated with the disease, our study highlights the negative effects of glucose-based ORT. Interestingly, in 2002, the WHO announced the recommendation to reduce the osmolarity in ORS [46]. Concomitantly, the final concentration of glucose was lowered from the initial 111 mM (WHO-ORS) to 75 mM (HYPO- ORS). Nevertheless, we observed that lowering the concentration of glucose in this range (from 111 mM glucose that was previously used, toward the 75 mM current recommendation, or even down to 50 mM, such as in our assays, because this concentration might better reflect the concentration within the intestine) did not reduce virulence gene expression in *V. cholerae* (Table 1). To our knowledge, this study is the first to describe the PTS sugar-dependent expression of virulence genes in *V. cholerae* cells grown in minimal salt conditions, although a putative cAMP-CRP binding site in the *tcpA* promoter region has been proposed previously [47] and the repression of TCP and CT by cAMP-CRP has been described [48]. Furthermore, a link between carbon catabolite repression and QS-dependent virulence repression has been reported before [33], [35], [49]; however, our data indicate that PTS-sugars also influence virulence expression in a QS-independent manner (Fig. S2), which might be due to the binding of the cAMP-CRP complex to the putative cAMP-CRP binding site [47] mentioned above.

In summary, our findings indicate that the virulence cascade of *V. cholerae* is inducible only in the presence of PTS sugars. Concomitantly, cholera toxin is produced on a larger scale when glucose is provided, which is a situation that most likely occurs when patients undergo standard glucose-based ORT. No such increase in CT production and virulence gene expression was observed when starch was used as alternative carbon source, which mimics rice-based ORT. Moreover, a switch from a glucose-based to a starch-based medium still resulted in decreased virulence gene expression in *V. cholerae*, which indicated that rice-based ORS would still be beneficial even if rice-based ORS were applied after the onset of symptoms. These results are supported by clinical studies, which demonstrated that rice-based ORS performed better in the field than standard glucose-based ORS [9]–[12]. The implementation of rice-based ORT has important effects on the epidemiological course of cholera. Indeed, the results of our model study of the Haitian cholera epidemic indicated a significant reduction in the total number of cholera cases using the range of parameters published for rice-based ORS. Notably, this significant reduction in cholera cases might even be an underestimation given that rice-based ORT might effectively down-regulate virulence gene expression in the pathogen thereby abolishing the hyperinfectivity state that has been described for *V. cholerae* [21], [50]. This would ultimately lead to an even lower transmission rate within the population. However, current recommendations by the WHO do not fully support the use of rice-based ORS for reasons such as product stability, increased packaging size, mode of application, and a three-fold cost over glucose-based ORS ($0.20 per liter of final ORS solution compared with $0.07) [51]. On the other hand, recent improvements in the food/nutrition industry might have optimized those handling- and cost-associated issues of starch-based products. Due to the described beneficial effects of rice-based ORS, the molecular explanations provided in this study, and the impact that those beneficial effects could have on epidemic progression, we recommend reconsidering starch-based ORS for cholera treatment.

## Supporting Information

Figure S1 **Relative expression of virulence-associated genes in indicated** ***V. cholerae*** **strains.** Virulence-expression was assessed in diverse *V. cholerae* isolates (**A**, O1 El Tor strain A1552; **B**, O1 El Tor strain N16961; **C**, O1 classical strain O395). Bacteria were grown with the indicated carbon sources and under virulence-non-inducing (−) and virulence-inducing (+) conditions. Different oligonucleotides were used for the amplification of El Tor-type *tcpA* or classical type *tcpA* (see Supporting Table S3). Statistics were applied using Student's t-test (* *P*≤0.05, ** *P*≤0.01, *** *P*≤0.001). All data represent averages from three independent biological replicates ± SD.
(PDF)

Figure S2 **Quorum sensing (QS)-dependent and QS-independent virulence gene expression.** (**A**) Comparison of virulence gene expression in QS-capable and QS-defective strains of *V. cholerae*. All strains were grown in the presence of the virulence inducer bicarbonate and glucose. (**B**) Virulence gene expression in the QS-defective strain ΔhapR that was grown with lactate or glucose as indicated. For expression details, see the legend of Supporting Fig. S1.
(PDF)

Figure S3 **Schematic representation of the model.** *S_i_* stands for susceptible individuals at node *i*, *I_S,i_* and *I_A,i_* for symptomatically and asymptomatically infected, respectively, *R_i_* for recovered and *B_i_* for bacterial concentration. Blue and red solid arrows indicate fluxes of individuals and bacteria, respectively, whereas the red dashed arrow indicates that the bacterial concentration governs the infection. Small blue forth and back arrows stand for human mobility between nodes and small red ones for hydrological *V. cholerae* transport. The single small blue arrow represents mortality due to cholera. Natural mortality is not shown.
(PDF)

Figure S4 **Evolution of the epidemics in Haiti per department.** Observed cases (grey bars), calibrated model (dark blue), and model runs with a ten percent reduction of symptomatic shedding rate as well as disease duration due to rice-based ORT (light blue). Shaded areas show the 95% confidence intervals resulting from parameter uncertainty; the solid lines show the median trajectory. The replacement of glucose-based with rice-based ORT was assumed to take place 30 days after the onset of the epidemic. Departments are (from left to right, top to bottom): Artibonite, Centre, Grande Anse, Nippes, Nord, Nord-Est, Nord-Ouest, Ouest, Sud, and Sud-Est. Daily rainfall in each department is also shown (top of each panel).
(PDF)

Figure S5 **Total cases through December 2011.** The data are based on the model, which was calibrated with different values of *q_A_*/*q_S_* (dark blue). Light blue bars show model runs using the exact same parameters except for the addition of a ten percent decrease of shedding rate and a ten percent shortening of the disease duration (see Supporting Text S1 for discussion).
(PDF)

Figure S6 **Evolution of the epidemics.** Observed cases (grey bars) and model calibrated with a range of different values for *q_A_*/*q_S_* (blue lines, see Supporting Fig. S5).
(PDF)

Figure S7 **Evolution of modeled susceptibles and symptomatic infected.** The solid lines show the modeled evolution of the number of total susceptibles over time as calibrated (dark blue) and with a 10% reduction in bacterial shedding rate as well as disease duration (light blue). Dashed lines: *idem* for the number of symptomatic infected. Trajectories shown correspond to the best performing parameter set. Note the higher number of susceptibles in fall 2011 after introducing the reductions, which leads to the more pronounced peak of total infections in November 2011.
(PDF)

Table S1 ***V. cholerae*** **strains used in this study.**
(DOCX)

Table S2 **Oligonucleotides used in this study.**
(DOCX)

Table S3 **Virulence gene expression under virulence-non-inducing (−) or virulence-inducing (+) conditions and in the presence of the indicated carbon sources.**
(DOCX)

Table S4 **Model parameters with their values and references.**
(DOCX)

Table S5 **Number of calibrated parameters and AIC scores for calibration with and without** ***q_A_*** **/** ***q_S_*** **.**
(DOCX)

Text S1 **Supporting methodology and results.** This file contains a description of the *in vitro* virulence gene expression approach and the qRT-PCR protocol. In addition, the file contains a description of the model and details with respect to the calibration of the model and the estimation of parameters. The references of all supporting methods, figures, and tables are also included in this file.
(PDF)
