# Supplementary figures and images for: Glucose- but Not Rice-Based Oral Rehydration Therapy Enhances the Production of Virulence Determinants in the Human Pathogen Vibrio cholerae
Source: PLoS Negl Trop Dis. 2014 Dec 4;8(12):e3347. doi: 10.1371/journal.pntd.0003347 (PMC4256474; doi:10.1371/journal.pntd.0003347)

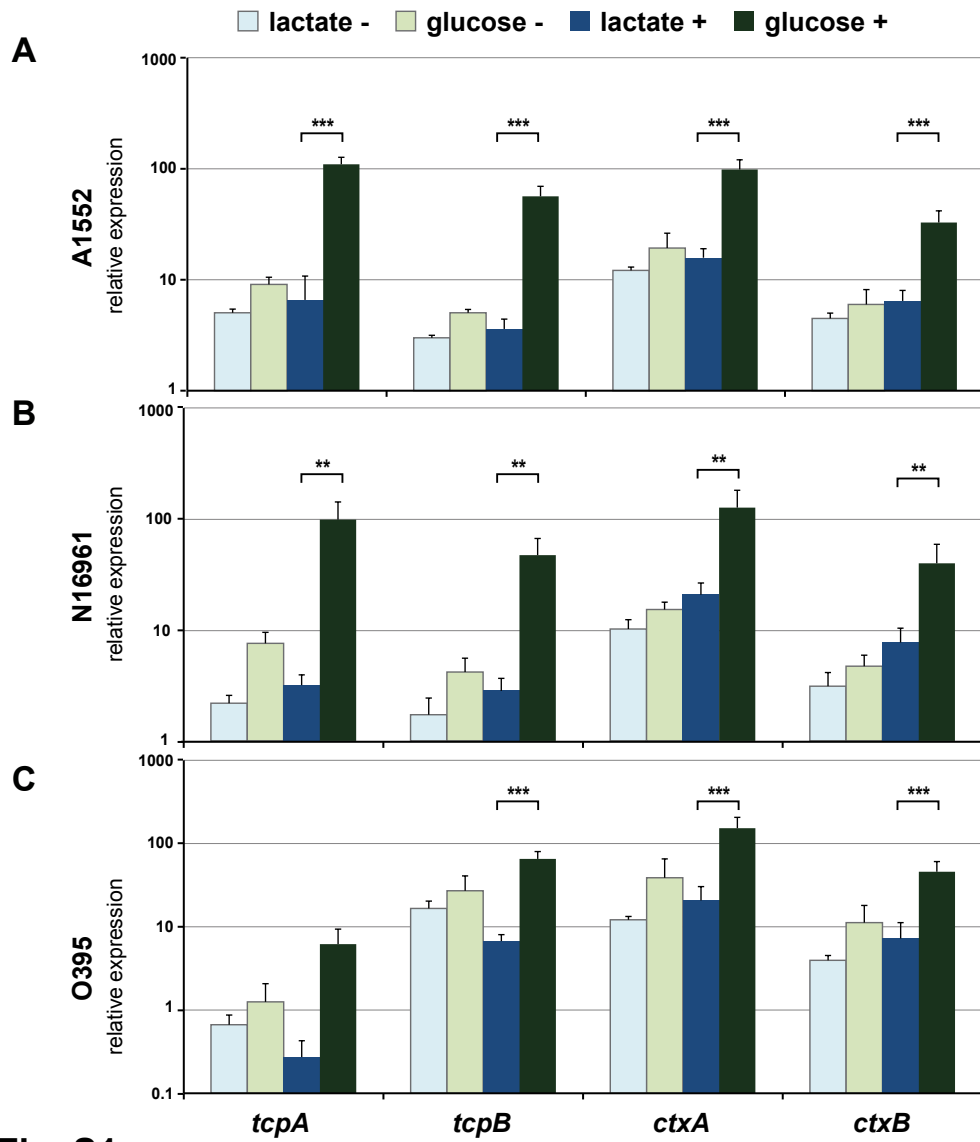

Fig. S1

Supplement: Figure S1 — Relative expression of virulence-associated genes in indicated V. cholerae strains. Virulence-expression was assessed in diverse V. cholerae isolates (A, O1 El Tor strain A1552; B, O1 El Tor strain N16961; C, O1 classical strain O395). Bacteria were grown with the indicated carbon sources and under virulence-non-inducing (−) and virulence-inducing (+) conditions. Different oligonucleotides were used for the amplification of El Tor-type tcpA or classical type tcpA (see Supporting Table S3). Statistics were applied using Student's t-test (* P≤0.05, ** P≤0.01, *** P≤0.001). All data represent averages from three independent biological replicates ± SD. (PDF) [file pntd.0003347.s001.pdf]

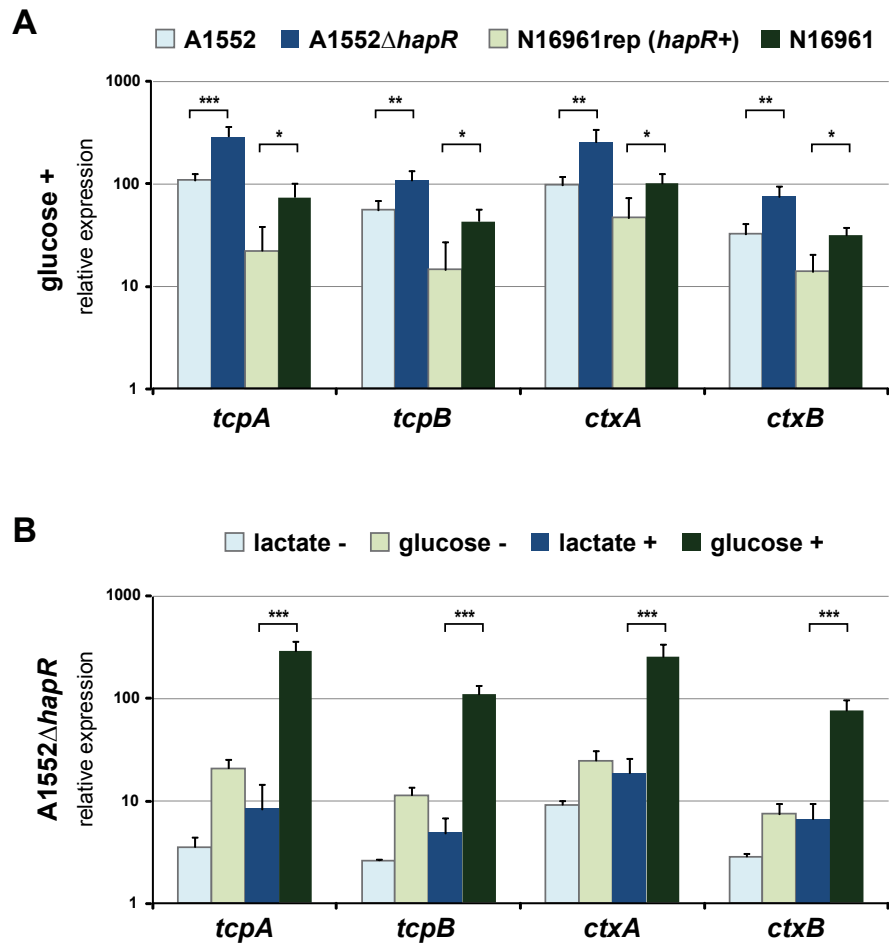

**Fig. S2**

Supplement: Figure S2 — Quorum sensing (QS)-dependent and QS-independent virulence gene expression. (A) Comparison of virulence gene expression in QS-capable and QS-defective strains of V. cholerae. All strains were grown in the presence of the virulence inducer bicarbonate and glucose. (B) Virulence gene expression in the QS-defective strain ΔhapR that was grown with lactate or glucose as indicated. For expression details, see the legend of Supporting Fig. S1. (PDF) [file pntd.0003347.s002.pdf]

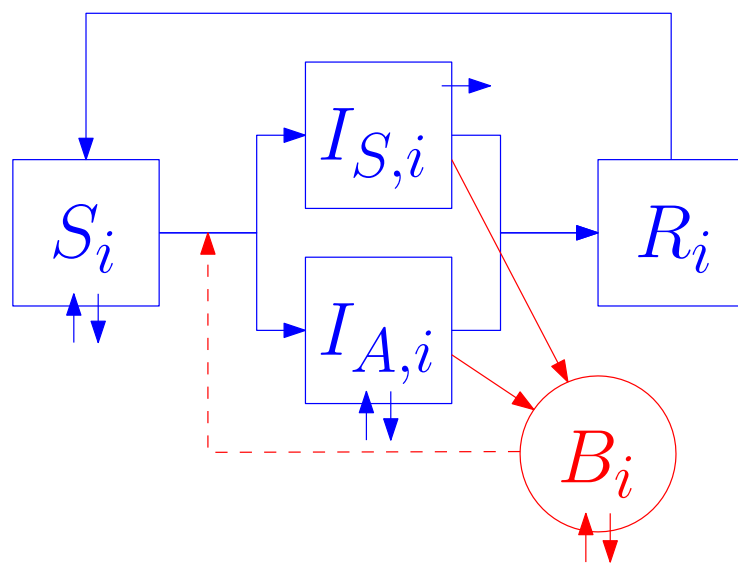

**Fig. S3**

Supplement: Figure S3 — Schematic representation of the model. Si stands for susceptible individuals at node i, IS,i and IA,i for symptomatically and asymptomatically infected, respectively, Ri for recovered and Bi for bacterial concentration. Blue and red solid arrows indicate fluxes of individuals and bacteria, respectively, whereas the red dashed arrow indicates that the bacterial concentration governs the infection. Small blue forth and back arrows stand for human mobility between nodes and small red ones for hydrological V. cholerae transport. The single small blue arrow represents mortality due to cholera. Natural mortality is not shown. (PDF) [file pntd.0003347.s003.pdf]

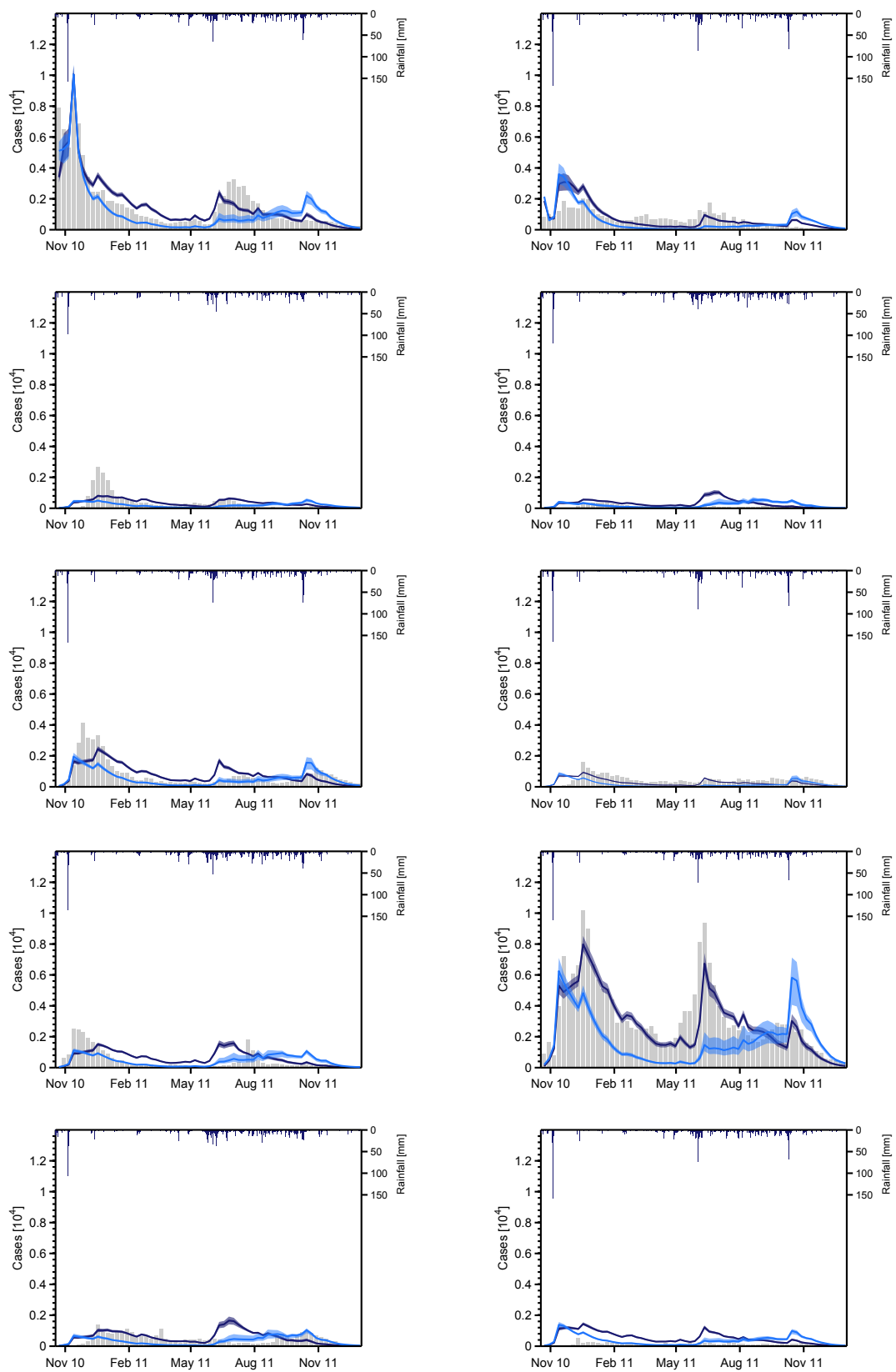

**Fig. S4**

Supplement: Figure S4 — Evolution of the epidemics in Haiti per department. Observed cases (grey bars), calibrated model (dark blue), and model runs with a ten percent reduction of symptomatic shedding rate as well as disease duration due to rice-based ORT (light blue). Shaded areas show the 95% confidence intervals resulting from parameter uncertainty; the solid lines show the median trajectory. The replacement of glucose-based with rice-based ORT was assumed to take place 30 days after the onset of the epidemic. Departments are (from left to right, top to bottom): Artibonite, Centre, Grande Anse, Nippes, Nord, Nord-Est, Nord-Ouest, Ouest, Sud, and Sud-Est. Daily rainfall in each department is also shown (top of each panel). (PDF) [file pntd.0003347.s004.pdf]

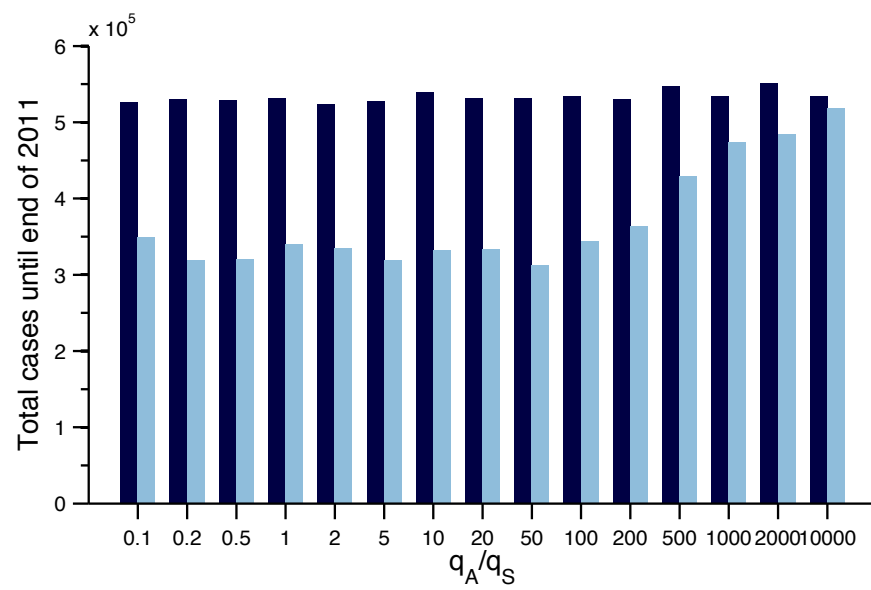

**Fig. S5**

Supplement: Figure S5 — Total cases through December 2011. The data are based on the model, which was calibrated with different values of qA/qS (dark blue). Light blue bars show model runs using the exact same parameters except for the addition of a ten percent decrease of shedding rate and a ten percent shortening of the disease duration (see Supporting Text S1 for discussion). (PDF) [file pntd.0003347.s005.pdf]

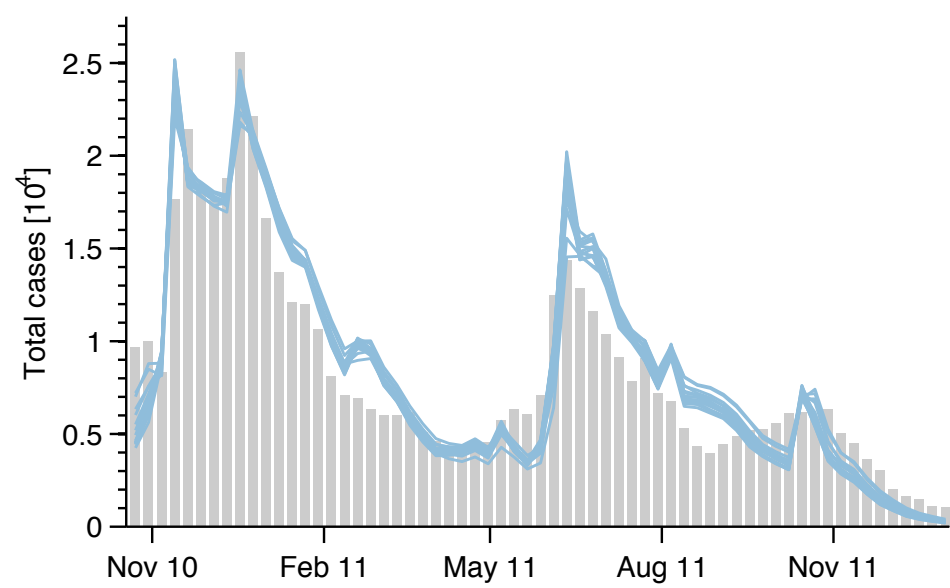

**Fig. S6**

Supplement: Figure S6 — Evolution of the epidemics. Observed cases (grey bars) and model calibrated with a range of different values for qA/qS (blue lines, see Supporting Fig. S5). (PDF) [file pntd.0003347.s006.pdf]

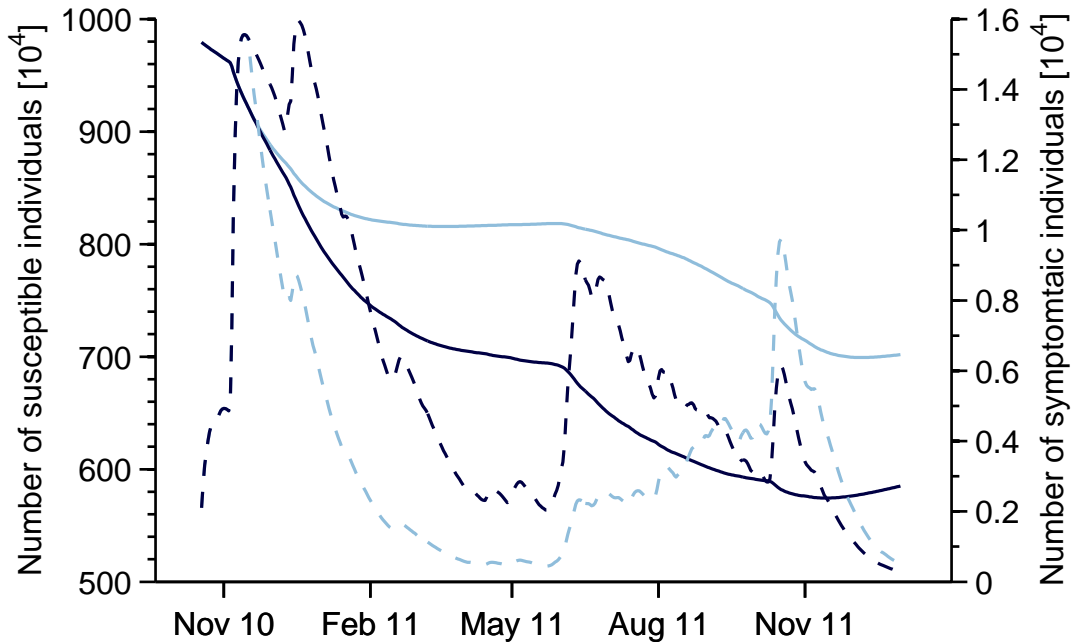

**Fig. S7**

Supplement: Figure S7 — Evolution of modeled susceptibles and symptomatic infected. The solid lines show the modeled evolution of the number of total susceptibles over time as calibrated (dark blue) and with a 10% reduction in bacterial shedding rate as well as disease duration (light blue). Dashed lines: idem for the number of symptomatic infected. Trajectories shown correspond to the best performing parameter set. Note the higher number of susceptibles in fall 2011 after introducing the reductions, which leads to the more pronounced peak of total infections in November 2011. (PDF) [file pntd.0003347.s007.pdf]
