# Supplementary material for: Glucose- but Not Rice-Based Oral Rehydration Therapy Enhances the Production of Virulence Determinants in the Human Pathogen Vibrio cholerae
Source: PLoS Negl Trop Dis. 2014 Dec 4;8(12):e3347. doi: 10.1371/journal.pntd.0003347 (PMC4256474; doi:10.1371/journal.pntd.0003347)
Supplement: Table S1 — V. cholerae strains used in this study. (DOCX) [file pntd.0003347.s008.docx]

**Table S1: *V. cholerae* strains used in this study.**

| **Strain** | **Genotype** | **Reference** |
| --- | --- | --- |
| A1552 (WT) | Wild type, O1 El Tor Inaba, Rif^R^ | [[38](#_ENREF_38)] |
| N16961 | Wild type, O1 El Tor Inaba, Strep^R^ | [[39](#_ENREF_39)] |
| O395 | Wild type, O1 classical, Strep^R^ | [[40](#_ENREF_40)] |
| N16961rep | N16961, *hapR*+::FRT | This study |
| A1552ΔhapR | A1552ΔVC0583, Rif^R^ | [[41](#_ENREF_41)] |
| A1552ΔscrA | A1552ΔVCA0653::FRT, Rif^R^ | [[42](#_ENREF_42)] |
| A1552ΔnagE | A1552ΔVC0995::FRT, Rif^R^ | This study |
| A1552ΔptsG | A1552ΔVC2013::FRT, Rif^R^ | This study |
| A1552-Tn*tfoX* | A1552-TntfoX, Rif^R^, Gent^R^ | [[4](#_ENREF_4)] |
| A1552ΔtcpP | A1552 ΔVC0826, Rif^R^ | This study |
| A1552ΔtcpP-Tn*tfoX* | A1552ΔVC0826-Tn*tfoX*, Rif^R^, Gent^R^ | This study |
| A1552ΔtoxR-Tn*tfoX* | A1552ΔVC0984::FRT-Tn*tfoX*, Rif^R^, Gent^R^ | This study |
| A1552ΔtoxT | A1552ΔVC0838, Rif^R^ | This study |
