# Supplementary material for: Glucose- but Not Rice-Based Oral Rehydration Therapy Enhances the Production of Virulence Determinants in the Human Pathogen Vibrio cholerae
Source: PLoS Negl Trop Dis. 2014 Dec 4;8(12):e3347. doi: 10.1371/journal.pntd.0003347 (PMC4256474; doi:10.1371/journal.pntd.0003347)
Supplement: Table S2 — Oligonucleotides used in this study. (DOCX) [file pntd.0003347.s009.docx]

**Table S2: Oligonucleotides used in this study.**

| **Primer name** [reference] | **Sequence (given in 5' to 3' direction)** |
| --- | --- |
| ***For TransFLP method (genetic manipulations)*** | |
| nagE FRT 1 | CCGTTGATGATTACCGCATGTTTCACAAGCACATC |
| nagE FRT 2 | GCTCCAGCCTACGCTTAGGTAGCATCAATGCTTTAC |
| nagE FRT 3 | GATGCTACCTAAGCGTAGGCTGGAGCTGCTTCGAA |
| nagE FRT 4 | GATGGCTTTCACATATGAATATCCTCCTTAGTTCCTATTC |
| nagE FRT 5 | GGAGGATATTCATATGTGAAAGCCATCAGCCCGAAAG |
| nagE FRT 6 | CAGAGGAGTAACACACCTCACCTGACCAATCAAAAC |
| ptsG_1 | TAACATTTAGGCTACGGGGGTATGC |
| ptsG_2 | AGCTCCAGCCTACGCTTAGAAAGTGGCGATCGCAGAACC |
| ptsG_3 | CCACTTTCTAAGCGTAGGCTGGAGCTGCTTCGAAGTTCC |
| ptsG_4 | AGCGATAGCCATATGAATATCCTCCTTAGT6TCCTATTCC |
| ptsG_5 | AAGGAGGATATTCATATGGCTATCGCTCACTGTGCTAAG |
| ptsG_6 | TACCGAAGATAGCTTGTACACCACC |
| ToxR_1 | TACTGCATCTGCTGGTGCACTACC |
| ToxR_2 | AGCTCCAGCCTACGCCTCTTTTGAGTTGTGTCCTAATCC |
| ToxR_3 | ACTCAAAAGAGGCGTAGGCTGGAGCTGCTTCGAAGTTCC |
| ToxR_4 | GCGAGAAATCATATGAATATCCTCCTTAGTTCCTATTCC |
| ToxR_5 | AGGAGGATATTCATATGATTTCTCGCAATGATTTGCATG |
| ToxR_6 | CTTTGATGGCATCGTTAGGGTTAGC |
| ***For construction of pGP704-28-SacB-ΔVC0826*** | |
| KO-VC0826-NcoI#1 | CGCCCATGGTTATCACGGAGTACTTCGTGATAATTAG |
| KO-VC0826#2 | GATAAATCCTTAGCGGACATACCCCATTACTTTACATTTTC |
| KO-VC0826#3 | TATGTCCGCTAAGGATTTATCAGCTCTGAAAGTCTAACTC |
| KO-VC0826-SacI#4 | CGCGAGCTCTAATGTCTTCTGTTCATAATTCACGG |
| ***For construction of pGP704Sac28ΔtoxT-II*** | |
| toxT-KO-1-NcoI | CGCCCATGGTAAGCACGGGTATACCAATCTGTGG |
| toxT-II-2 | ACATAAATAATTACCCAATCATTGCGTTCTACTCTGAAG |
| toxT-II-3 | CAATGATTGGGTAATTATTTATGTTGACAGGAGTTGCAG |
| toxT-II-4-XbaI | CGCTCTAGATCACATAGGATATGATGAAAGCCGC |
| ***For qRT-PCR (expression analysis)*** | |
| qRT ctxB for | CGTATACAGAATCTCTAGCTG |
| qRT ctxB rev | CAATCCTCAGGGTATCCTTC |
| qRT ctxA for | GATGGATATGTTTCCACCTC |
| qRT ctxA rev | GAGGACTGTATGCCCCTAAT |
| qRT tcpA for [[43](#_ENREF_43)] | GAATATGACTAAGGCTGCGC |
| qRT tcpA rev [[43](#_ENREF_43)] | GCTGAAACCTTACCAAGGCT |
| qRT tcpAc for  (specific for classical strains) | GCCGCGCAAAGTCTCAATAG |
| qRT tcpAc rev  (specific for classical strains) | GGGTTTTTTGCCTCATCGGA |
| qRT_tcpB_fwd [[43](#_ENREF_43)] | TCAGTCTTGCCCAAACCGGA |
| qRT_tcpB_bwd [[43](#_ENREF_43)] | ATAGGCCTTTCGCACTGACC |
| qRT_toxT_fwd [[43](#_ENREF_43)] | TAC GCG TAA TTG GCG TTG GG |
| qRT_toxT_bwd [[43](#_ENREF_43)] | ACG CTA GCA AAC CCA GAC TG |
| gyrA-157-fwd [[4](#_ENREF_4)] | AATGTGCTGGGCAACGACTG |
| gyrA_332_bwd [[4](#_ENREF_4)] | GAGCCAAAGTTACCTTGGCC |
