# Supplementary material for: Glucose- but Not Rice-Based Oral Rehydration Therapy Enhances the Production of Virulence Determinants in the Human Pathogen Vibrio cholerae
Source: PLoS Negl Trop Dis. 2014 Dec 4;8(12):e3347. doi: 10.1371/journal.pntd.0003347 (PMC4256474; doi:10.1371/journal.pntd.0003347)
Supplement: Table S3 — Virulence gene expression under virulence-non-inducing (−) or virulence-inducing (+) conditions and in the presence of the indicated carbon sources. (DOCX) [file pntd.0003347.s010.docx]

**Table S3: Virulence gene expression under virulence-non-inducing (-) or virulence-inducing (+) conditions and in the presence of the indicated carbon sources.**

| **virulence genes** | **lac** | | **fold induction** | **glc** | | **fold induction** | **fold difference glc (+) / lac (+)** |
| --- | --- | --- | --- | --- | --- | --- | --- |
|  | - | + |  | - | + |  |  |
| **A1552 (*V. cholerae* El Tor, South America 1992)** | | | | | | | |
| *tcpA* | 5.03 | 6.57 | 1.3 | 9.07 | 109.74 | 12.1 | 16.7 |
| *tcpB* | 2.99 | 3.60 | 1.2 | 5.03 | 56.37 | 11.2 | 15.7 |
| *ctxA* | 12.13 | 15.75 | 1.3 | 19.26 | 98.46 | 5.1 | 6.2 |
| *ctxB* | 4.47 | 6.43 | 1.4 | 5.98 | 32.72 | 5.5 | 5.1 |
| **N16961 (*V. cholerae* El Tor, Bangladesh, 1971)** | | | | | | | |
| *tcpA* | 2.18 | 3.18 | 1.5 | 7.57 | 98.97 | 13.1 | 31.1 |
| *tcpB* | 1.72 | 2.82 | 1.6 | 4.18 | 47.20 | 11.3 | 16.7 |
| *ctxA* | 10.20 | 20.84 | 2.0 | 15.31 | 126.71 | 8.3 | 6.1 |
| *ctxB* | 3.10 | 7.72 | 2.5 | 4.71 | 39.89 | 8.5 | 5.2 |
| **O395 (*V. cholerae* classical, India, 1965)** | | | | | | | |
| *tcpA* | 0.66 | 0.27 | 0.4 | 1.25 | 6.18 | 4.9 | 22.7 |
| *tcpB* | 16.60 | 6.73 | 0.4 | 27.08 | 65.51 | 2.4 | 9.7 |
| *ctxA* | 12.13 | 20.70 | 1.7 | 38.81 | 153.35 | 4.0 | 7.4 |
| *ctxB* | 3.95 | 7.25 | 1.8 | 11.24 | 45.98 | 4.1 | 6.3 |
