# Supplementary material for: Glucose- but Not Rice-Based Oral Rehydration Therapy Enhances the Production of Virulence Determinants in the Human Pathogen Vibrio cholerae
Source: PLoS Negl Trop Dis. 2014 Dec 4;8(12):e3347. doi: 10.1371/journal.pntd.0003347 (PMC4256474; doi:10.1371/journal.pntd.0003347)
Supplement: Table S4 — Model parameters with their values and references. (DOCX) [file pntd.0003347.s011.docx]

**Table S4: Model parameters with their values and references.** Parameters in the upper part of the table have been taken from the literature or estimated. Calibration parameters (with 95% confidence intervals) are shown in the lower part of the table.

| **Parameter** | **Units** | **Value** | **Reference** |  |
| --- | --- | --- | --- | --- |
| $\beta$ | $\text{day}^{-1}$ | $1.0$ | [[5](#_ENREF_5),[6](#_ENREF_6),[44-46](#_ENREF_44)] | |
| $\alpha$ | $\text{day}^{-1}$ | $4.0\times{10}^{-3}$ | [[5](#_ENREF_5)] PAHO, 2011 | |
| $\mu$ | $\text{day}^{-1}$ | $1/(61\times365)$ | [[5](#_ENREF_5)] CIA, 2009 | |
| $\gamma$ | $\text{day}^{-1}$ | $0.2$ | [[5](#_ENREF_5),[6](#_ENREF_6),[19](#_ENREF_19),[29](#_ENREF_29),[44](#_ENREF_44),[45](#_ENREF_45)] | |
| $\mu_{B}$ | $\text{day}^{-1}$ | $0.2$ | [[5](#_ENREF_5),[6](#_ENREF_6),[19](#_ENREF_19),[44](#_ENREF_44),[45](#_ENREF_45)] | |
| $\rho$ | - | $1/(3\times365)$ | [[5](#_ENREF_5),[47](#_ENREF_47)] | |
| $\varepsilon$ | - | 5 | [[9](#_ENREF_9)] | |
| $p_{A}/p_{S}$ | - | ${10}^{-3}$ | [[9](#_ENREF_9),[29](#_ENREF_29),[47](#_ENREF_47)] | |
| $q_{A}/q_{S}$ | - | $200 ({10}^{-1}\text{to}{10}^{4})$ | (see Supporting Text S1 and Supporting Fig. S6) | |
| $\theta$ | $\text{day}^{-1}$ | 0.55 [0.49 0.63] | - | |
| $l$ | $\text{day}^{-1}$ | 0.20 [0.14 0.24] | - | |
| $m$ | - | 0.037 [0.026 0.050] | - | |
| $D$ | $\text{km}$ | 343 [221 398] | - | |
| $\phi$ | $\text{day mm}^{-1}$ | 0.081 [0.073 0.094] | - | |
| $\sigma$ | - | 0.10 [0.10 0.11] | - | |
