# Supplementary material for: Glucose- but Not Rice-Based Oral Rehydration Therapy Enhances the Production of Virulence Determinants in the Human Pathogen Vibrio cholerae
Source: PLoS Negl Trop Dis. 2014 Dec 4;8(12):e3347. doi: 10.1371/journal.pntd.0003347 (PMC4256474; doi:10.1371/journal.pntd.0003347)
Supplement: Table S5 — Number of calibrated parameters and AIC scores for calibration with and without qA / qS . (DOCX) [file pntd.0003347.s012.docx]

**Table S5: Number of calibrated parameters and AIC scores for calibration with and without** $\boldsymbol{q}_{\boldsymbol{A}}\boldsymbol{/}\boldsymbol{q}_{\boldsymbol{S}}$ **(for details see text).** The last column shows the Akaike difference, which must be > 4 for significance.

| **Model** | **n. of parameters** | **AIC** | **ΔAIC** |
| --- | --- | --- | --- |
| calibration with $q_{A}/q_{S} =200$ | 6 | 12205 | - |
| calibrating also $q_{A}/q_{S}$ | 7 | 12217 | 12 |
