# Supplementary material for: Glucose- but Not Rice-Based Oral Rehydration Therapy Enhances the Production of Virulence Determinants in the Human Pathogen Vibrio cholerae
Source: PLoS Negl Trop Dis. 2014 Dec 4;8(12):e3347. doi: 10.1371/journal.pntd.0003347 (PMC4256474; doi:10.1371/journal.pntd.0003347)
Supplement: Text S1 — Supporting methodology and results. This file contains a description of the in vitro virulence gene expression approach and the qRT-PCR protocol. In addition, the file contains a description of the model and details with respect to the calibration of the model and the estimation of parameters. The references of all supporting methods, figures, and tables are also included in this file. (PDF) [file pntd.0003347.s013.pdf]

Text S1

Supporting Methodology and Results

Induction of virulence gene expression

V. cholerae strains were grown overnight in M9 minimal medium (Sigma-Aldrich, Buchs, Switzerland) supplemented with vitamins (MEM, Gibco), Bacto casamino acids (Becton, Dickinson and Company) and glucose. Overnight cultures were washed in minimal medium and diluted 1:100 in M9 medium containing vitamins and casamino acids, as well as the indicated carbon source. Glucose, N-Acetylglucosamine (GlcNAc), sucrose, galactose, lactate, or succinate (all purchased from Sigma-Aldrich, Buchs, Switzerland) served as carbon sources and were provided at a concentration of 50 mM. Alternatively, starch from potato or from rice (Sigma-Aldrich, Buchs, Switzerland) was boiled for 10 min before being mixed with the M9 medium components to a final concentration of 10 g/l to mimic starch-based oral rehydration conditions.

The strains were grown at 37 °C under shaking conditions until the bacteria reached an optical density at 600 nm (OD_{600}) of ~0.1. At that time, sodium bicarbonate (Sigma-Aldrich/Fluka, Buchs, Switzerland), was added to a final concentration of 100 mM to induce virulence [1]. Samples for expression analysis (qRT-PCR) were harvested when the culture reached an OD_{600} of ~0.8-1.0, whereas samples for the quantification of cholera toxin (ELISA) were taken 5 h post-bicarbonate addition. Because the rice-based starch changed the turbidity of the medium, bacteria were enumerated using a Neubauer chamber instead of relying on optical density measurements, and the latter was back calculated accordingly. For inhibition of ToxT by the small-molecule virstatin [2,3] the bacteria were grown either in the presence of DMSO (vehicle control) or 50µM virstatin (dissolved in DMSO; Enzo Life Sciences, Inc.).

Quantitative reverse transcription-based PCR (qRT-PCR)

Bacterial cells were harvested from 5 ml of culture and lysed in 1 ml Tri Reagent (Sigma-Aldrich, Buchs, Switzerland) or 1 ml TRIzol (Invitrogen, Basel). The samples were stored at -80 °C until RNA isolation was performed using GenElute™ Mammalian Total RNA Miniprep Kit (Sigma-Aldrich, Buchs, Switzerland). Putative contaminating DNA was removed by a TURBO DNA-free™ DNase treatment (Ambion). The reverse transcription of 1 µg of total RNA was performed using a Transcriptor Universal cDNA Master mix (Roche, Rotkreuz, Switzerland). Quantitative PCR was performed as described previously [4] with expression values given relative to the reference gene *gyrA*.

Model description

Our spatially explicit epidemiological model builds on the model presented by Rinaldo *et al.* [5]. The epidemiological dynamics are modeled using a system of differential equations, taking into account hydrological pathogen transport, human mobility as well as precipitation:

$$\begin{aligned}
\frac{dS_i}{dt} &= \mu(H_i - S_i) - \mathcal{F}_i(t)S_i + \rho R_i \\
\frac{dI_{S,i}}{dt} &= \sigma\mathcal{F}_i(t)S_i - (\gamma + \mu + \alpha)I_{S,i} \\
\frac{dI_{A,i}}{dt} &= (1-\sigma)\,\mathcal{F}_i(t)S_i - (\varepsilon\gamma + \mu)I_{A,i} \\
\frac{dR_i}{dt} &= \gamma\left(I_{S,i} + \varepsilon I_{A,i}\right) - (\rho + \mu)R_i \\
\frac{dB_i}{dt} &= -\mu_B B_i - l\left(B_i - \sum_{j=1}^{n} P_{ji}\frac{W_j}{W_i}B_j\right) + \frac{1}{W_i}[1 + \phi J_i(t)]\left(p_S q_S \mathcal{G}_{S,i}(t) + p_A q_A \mathcal{G}_{A,i}(t)\right).
\end{aligned} \tag{1}$$

Individuals living at node i can be susceptible (S_i), symptomatically infected ($I_{S,i}$), asymptomatically infected ($I_{A,i}$) or recovered (R_i). B_i represents the bacterial concentration in the water reservoir of the node. The population is assumed to be in demographic equilibrium, with a constant recruitment μH_i of susceptibles, where μ is the mortality rate (unrelated to cholera) and H_i is the total population size. Note that the partition of the infected compartment into two classes is employed for the first time in a spatially explicit model here.

Susceptible individuals are infected at rate:

$$\mathcal{F}_i(t) = \beta\left[(1-m)\frac{B_i}{K + B_i} + m\sum_{j=1}^{n} Q_{ij}\frac{B_j}{K + B_j}\right], \tag{2}$$

where β stands for the maximum rate of infection. $B_i/(K + B_i)$ is the probability of infection after exposure to concentration B_i, with K being the half-saturation constant [6,7]. This is modeled assuming that the force of infection in a given node depends on the local concentration B_i for a fraction $(1 - m)$ of the susceptible hosts and on the concentration B_j of the surrounding communities for the remaining fraction m. The parameter m represents the community-level probability that individuals travel outside

their node and it is assumed, in this formulation, to be node-independent. The concentrations B_j are weighted according to the probabilities Q_{ij} that an individual living in node i would reach j as a destination [8]:

$$Q_{ij} = \frac{H_j e^{-d_{ij}/D}}{\sum_{k\neq i}^{n} H_k\, e^{-d_{ik}/D}}, \qquad (3)$$

where the attractiveness of node j is governed by its population size. However, deterrence exponentially increases with distance d_{ij}between nodes i and j according to a shape factor D.

Infected individuals show symptoms with probability σ. These individuals may recover at rate γ, die from cholera or from other causes at rate α or μ, respectively. Asymptomatically infected individuals do not die from cholera and recover faster by a factor ε [9]. Recovered individuals lose their acquired immunity at rate ρ, or they die at rate μ.

The bacterial concentration at each node is supplied by the product of the bacterial shedding rate p of the infected individuals and the probability q that the freshly shed bacteria reach the water reservoir, divided by the volume of the later. This volume is considered to be directly proportional to the population size at the node $W_i = cH_i$. Parameters p and q take different values for symptomatic (subscript S) and asymptomatic (subscript A) individuals because people without symptoms shed less bacteria [9] and because of possibly different sanitation conditions. See section *Model calibration and parameter estimation* below for a thorough discussion of these aspects. Symptomatic individuals are assumed to stay within their home node, whereas the movement of asymptomatic individuals is modeled similar to equation (2).
Asymptomatic people leave their node j with probability m and reach node i based on the gravity factor Q_{ji}:

$$\mathcal{G}_{S,i} = I_{S,i}$$
$$\mathcal{G}_{A,i} = (1-m)I_{A,i} + m\sum_{j=1}^{n} Q_{ji} I_{A,i}\,. \qquad (4)$$

We further considered that rain events cause a deterioration of sanitation conditions and a higher probability of the contamination of water reservoirs [10]. The shedding term in equation (1) thus increases with precipitation intensity $J_i(t)$according to the factor ϕ [5]. In addition, *V. cholerae*

pathogens are subject to natural mortality at rate μ_B and hydrological transport at rate l. In our case, the probability P_{ij} that pathogens travel from node i to j is equal to one if j is the downstream nearest neighbor of i, and zero if otherwise.

Modeling the Haiti epidemic

In order to apply the above model to the Haitian epidemics we used the countries road network (© OpenStreetMap contributors, available online at http://www.openstreetmap.org under the Open Database License) as well as the hydrological network, which was derived from a digital terrain model (DTM) (data available from the U.S. Geological Survey, http://earthexplorer.usgs.gov) using established hydrological methods [11-15]. The procedure consists in the determination of the unique steepest descent flow path from every pixel of the DTM to the sea. Pixels draining to the one and the same outlet belong to the same river basin. Because this leads to a very heterogeneous basin size distribution, the larger watersheds had to be split into smaller units according to catchment divides, whereas the coastal (smaller) watersheds were aggregated to reasonable size. Using this procedure, the Haitian territory was subdivided into 365 hydrological subunits, which each correspond to a node in our model. Nodes are connected through the hydrological network and to the road network. From the latter, the shortest distance between each pair of nodes was calculated [16] and taken as an input to build the distance matrix that was used in equation (3). The population of each subunit (see Fig. 5 in the main text) was derived from a remotely sensed population distribution (Oak Ridge National Laboratory, 2011; http://ornl.gov/sci/landscan/index.shtml), which has been updated to correspond to the most recent population estimates [17]. Daily precipitation fields were obtained from a remotely sensed dataset by the National Aeronautics and Space Agency (NASA), which has a spatial resolution of 0.25 latitude and longitude [18]. We assume that, at the beginning of the epidemics, the entire population was susceptible ($S_i(t_0) = H_i$)[5,19-23]. As an initial condition, we introduced a number of infected individuals to selected nodes in the Centre and in the Artibonite departments of Haiti according to a detailed report about the state of the epidemics on 20 October 2010 [21]. Additionally, an initial equilibrium bacterial concentration ($B_i(t_0) = (p_S q_S I_{S,i}(t_0) + p_A q_A I_{A,i}(t_0))$) was imposed at the same nodes. We further assumed that no recovered individuals were present at the beginning of the epidemics ($R_i(t_0) = 0$).

Model calibration and parameter estimation

To reduce the number of unknown parameters we introduced the adimensional bacterial concentration $\mathcal{B}_i = B_i/K$ along with the aggregated contamination rate for symptomatics $\theta = p_S q_S/cK$. Parameters that could not be derived from previous work or from the literature were calibrated using data from daily epidemiological reports that are available on the website of the Public Ministry of Health of Haiti (Ministère de la Santé Publique et de la Population, http://mspp.gouv.ht/) [24]. The calibration period starts at the beginning of the epidemics (20 October 2010) and ends in December 2011. Because the data are freely available at the department level only, our model outputs needed to be upscaled for comparison. We assumed that the number of weekly reported cases corresponds to the number of newly infected symptomatic individuals. To derive the number of reported cases from the model, one thus needs to calculate I_i (the modeled newly reported cases) as follows:

$$I_i(t) = \int_t^{t+\Delta t} \sigma \mathcal{F}_i(t) S_i \, \mathrm{d}t \ , \qquad (5)$$

where Δt is equal to one week. The sum of the squared residuals between the reported cases and the model output that was calculated according to (5) was used as the objective function. For calibration, we relied on a Markov Chain Monte Carlo (MCMC) approach with several chains, which the so called differential evolution adaptive Metropolis ($DREAM_{ZS}$) algorithm [25,26]. The parameters that related to rice-based ORS (τ_1 and τ_2), which are described in section *Modeling the effects of rice-based ORS*, have been set to zero during calibration because we assume that only standard (glucose-based) ORS has been used. See Supporting Table S4 for other parameter values and references.

Modeling the effect of rice-based ORS

Our model can be used to estimate the impact of the reduction of the disease duration and of the stool volume on the overall dynamics of the epidemics in space and time. To include these effects, we assume that the reported cases correspond to the symptomatically infected and that all of these individuals received at least basic treatment with ORS (in a hospital, at home or at a so called rehydration point [27]). In addition, we assume that the reduction in stool volume correlates with a reduction in the number of *V. cholerae* that was shed per unit time. The effects can then be integrated into our model by reducing the disease duration as well as the bacterial shedding rate for symptomatic individuals. Two additional adjustable parameters (τ_1 and τ_2) have thus been added to our model:

$$\begin{aligned}
\frac{dSi}{dt} &= \mu(H_i - S_i) - \mathcal{F}_i(t)S_i + \rho R_i \\
\frac{dI_{S,i}}{dt} &= \sigma\mathcal{F}_i(t)S_i - \left(\left(\frac{1}{1-\tau_1}\right)\gamma + \mu + \alpha\right)I_{S,i} \\
\frac{dI_{A,i}}{dt} &= (1-\sigma)\,\mathcal{F}_i(t)S_i - (\varepsilon\gamma + \mu)I_{A,i} \\
\frac{dR_i}{dt} &= \gamma\left(\left(\frac{1}{1-\tau_1}\right)I_{S,i} + \varepsilon I_{A,i}\right) - (\rho + \mu)R_i \\
\frac{dB_i}{dt} &= -\mu_B B_i - l\left(B_i - \sum_{j=1}^{n} P_{ji}\frac{W_j}{W_i}B_j\right) + \frac{p_S q_S}{W_i}[1 + \phi J_i(t)]\left((1-\tau_2)\mathcal{G}_{S,i}(t) + \frac{p_A q_A}{p_S q_S}\mathcal{G}_{A,i}(t)\right).
\end{aligned} \qquad (6)$$

These parameters can be used to adapt the recovery rate as well as the bacterial shedding rate of the symptomatic individuals only. τ_1 is the reduction in the disease duration and τ_2 is the reduction in the bacterial shedding rate, which are both expressed as a fraction of one. For calibration, both parameters have been fixed to zero (no effect), thus the equations were equivalent to (1).

Whereas values for the ratio of asymptomatic to symptomatic shedding rates (p_A/p_S) are available in the literature [9,28,29], the relative probability that asymptomatics contaminate environmental water bodies (q_A/q_S) is difficult to estimate. Assuming that a high fraction of symptomatic individuals is admitted to hospitals or treatment centers, this ratio indeed depends on the sanitary conditions in health care facilities compared with regular households. One would expect sanitation to be a key issue in hospitals. However, as few functional sewer systems exist in Haiti [30], few options are left for proper disposal of human fecal matter. In addition, during the peak phases of the epidemics, health care facilities were subject to over-occupancy [27]. Furthermore, during cholera outbreaks, symptomatic patients are released as soon as their condition starts improving (less than three liquid stools in six hours [31]), even if these individuals might still shed *V. cholerae*. Therefore, depending on the relative impact of the various effects that are stated above, the ratio (q_A/q_S) can take a high range of values. By adding this ratio as a parameter to the calibration procedure, we were not able to identify its value uniquely due to a very flat posterior distribution. This is probably due to its high correlation with other parameters (e.g. θ). In addition, the Akaike information criterion (AIC) [32,33] indicates that the improvements of the model output are not significant (see Supporting Table S5). Thus, we decided not to calibrate q_A/q_S but to proceed as follows: to assess the sensitivity of the remaining calibration parameters as well as the model outputs with respect to changes of the ratio q_A/q_S, we calibrated the model with a range of different values. From the Supporting Figure S6, it can be seen that we are able to calibrate the model almost equally well independently of the value of q_A/q_S, whereas the values of the calibrated parameters vary. We note that θ is particularly sensitive to changes in q_A/q_S because

both parameters directly affect bacterial shedding and they thus compensate. Supporting Figure S5 shows the important influence that q_A/q_S has on the result of a ten percent reduction of the bacterial shedding rate as well as the disease duration (using parameters τ_1 and τ_2). This influence due to the fact that the ratio q_A/q_S acts directly on the relative contribution of symptomatics versus asymptomatics to the environmental bacteria concentration, whereas only symptomatics are treated with ORS. It can further be seen that only a very high value of q_A/q_S (above 200) significantly reduces the impact of rice-based ORS. Such values would imply that the probability of contaminating the environmental water bodies is more than 200 times higher for asymptomatically than for symptomatically infected individuals. Based on the above analysis, it is unlikely that this fraction takes values as high as that under Haitian conditions. Thus, we assume the conservative value of $q_A/q_S = 200$ for the purpose of this study. To show the influence of a given treatment effect on the overall disease dynamics, τ_1 and τ_2 were set to appropriate values after an initial phase of 30 days, which we considered necessary to initiate systematic treatment with rice-based ORS. The calibrated parameters remained fixed.

The values taken by these parameters vary according to different sources [34-37]. Gore *et al.* [34] conducted a comparative analysis of several studies and their results indicate that the reduction in disease duration varies around 12% (5 to 19%) and the reduction of stool volume during the first 24h around 36% (28 to 44%) for adults (when rice-based ORT was compared with glucose-based WHO-ORS). Please note that whereas the reduction of disease duration can be directly related to parameter τ_1, the reduction of stool volume would only correspond to parameter τ_2 if the bacterial concentration in the stool remained constant. As this hypothesis is unlikely to be verified, and bacterial concentration in the stool is hardly ever reported, we conducted our analysis using a broad range of values for $\tau_1$and τ_2.

The effect on the epidemiological dynamics of setting both treatment parameters (τ_1 and τ_2) to a hypothetical value of 0.1, which assumes a ten percent reduction of disease duration and shedding rate for symptomatically infected individuals, can be seen in Figure 5, and Supporting Figures S4-S6. Note that the reduction has only been applied after an initial period of 30 days. One can clearly state a considerable reduction in disease incidence in all departments that begins at the moment the reduction is applied. However, it can be seen that the model predicts a higher number of cases during fall 2011, most likely due to a high number of susceptibles in the system during that period (Figure S7). Overall, as discussed in the main text, the assumption of maintaining other infection processes unchanged by the

introduction of alternative hydration treatments seems quite reasonable, thus allowing us to issue predictive scenarios to the unfolding epidemic patterns.

Supporting References

1. Nielsen AT, Dolganov NA, Rasmussen T, Otto G, Miller MC, et al. (2010) A bistable switch and anatomical site control *Vibrio cholerae* virulence gene expression in the intestine. PLoS Pathog 6: e1001102.
2. Hung DT, Shakhnovich EA, Pierson E, Mekalanos JJ (2005) Small-molecule inhibitor of *Vibrio cholerae* virulence and intestinal colonization. Science 310: 670-674.
3. Shakhnovich EA, Hung DT, Pierson E, Lee K, Mekalanos JJ (2007) Virstatin inhibits dimerization of the transcriptional activator ToxT. Proc Natl Acad Sci U S A 104: 2372-2377.
4. Lo Scrudato M, Blokesch M (2012) The regulatory network of natural competence and transformation of *Vibrio cholerae*. PLoS Genet 8: e1002778.
5. Rinaldo A, Bertuzzo E, Mari L, Righetto L, Blokesch M, et al. (2012) Reassessment of the 2010-2011 Haiti cholera outbreak and rainfall-driven multiseason projections. Proc Natl Acad Sci USA 109: 6602-6607.
6. Codeco CT (2001) Endemic and epidemic dynamics of cholera: the role of the aquatic reservoir. BMC Infect Dis 1: 1.
7. Capasso V, Paveri-Fontana SL (1979) A mathematical model for the 1973 cholera epidemic in the European Mediterranean region. Rev Epidemiol Sante Publique 27: 121-132.
8. Erlander S, Stewart NF (1990) The Gravity Model in Transportation Analysis - Theory And Extensions. Utrecht, The Netherlands: VSP.
9. Nelson EJ, Harris JB, Morris JG, Jr., Calderwood SB, Camilli A (2009) Cholera transmission: the host, pathogen and bacteriophage dynamic. Nat Rev Microbiol 7: 693-702.
10. Gaudart J, Rebaudet S, Barrais R, Boncy J, Faucher B, et al. (2013) Spatio-temporal dynamics of cholera during the first year of the epidemic in Haiti. PLoS Negl Trop Dis 7: e2145.
11. Band LE (1986) Topographic Partition of Watersheds with Digital Elevation Models. Water Resour Res 22: 15-24.
12. Montgomery DR, Dietrich WE (1998) Where do channels begin? Nature 336: 232 - 234.
13. Montgomery DR, Dietrich WE (1992) Channel initiation and the problem of landscape scale. Science 255: 826-830.

14. Tarboton DG (1997) A new method for the determination of flow directions and upslope areas in grid digital elevation models. Water Resour Res 33: 309-319.

15. Rodriguez-Iturbe I, Rinaldo A (2001) Fractal River Basins: Chance and Self-Organization. Cambridge University Press.

16. Dijkstra EW (1959) A note on two problems in connexion with graphs. Numerische Mathematik 1: 269-271.

17. Mukandavire Z, Smith DL, Morris JG, Jr. (2013) Cholera in Haiti: reproductive numbers and vaccination coverage estimates. Sci Rep 3: 997.

18. Huffman GJ, Adler RF, Bolvin DT, Nelkin EJ (2010) The TRMM Multi-Satellite Precipitation Analysis (TMPA). In: Gebremichael M, Hossain F, editors. Satellite Rainfall Applications for Surface Hydrology: Springer Netherlands. pp. 3–22.

19. Bertuzzo E, Mari L, Righetto L, Gatto M, Casagrandi R, et al. (2011) Prediction of the spatial evolution and effects of control measures for the unfolding Haiti cholera outbreak. Geophys Res Lett 38: doi:10.1029/2011GL046823.

20. Walton DA, Ivers LC (2011) Responding to cholera in post-earthquake Haiti. N Engl J Med 364: 3-5.

21. Piarroux R, Barrais R, Faucher B, Haus R, Piarroux M, et al. (2011) Understanding the cholera epidemic, Haiti. Emerg Infect Dis 17: 1161-1168.

22. Sack DA (2011) How many cholera deaths can be averted in Haiti? Lancet.

23. Enserink M (2010) Infectious diseases. Haiti's outbreak is latest in cholera's new global assault. Science 330: 738-739.

24. Barzilay EJ, Schaad N, Magloire R, Mung KS, Boncy J, et al. (2013) Cholera surveillance during the Haiti epidemic--the first 2 years. N Engl J Med 368: 599-609.

25. Vrugt JA, ter Braak CJF, Clark MP, Hyman JM, Robinson BA (2008) Treatment of input uncertainty in hydrologic modeling: Doing hydrology backward with Markov chain Monte Carlo simulation. Water Resources Research 44: W00B09.

26. Vrugt JA, ter Braak CJF, Diks CGH, Robinson BA, Hyman JM, et al. (2009) Accelerating Markov Chain Monte Carlo Simulation by Differential Evolution with Self-Adaptive Randomized Subspace Sampling. Int J Nonlin Sci Num 10: 273-290.

27. Walton D, Suri A, Farmer P (2011) Cholera in Haiti: Fully Integrating Prevention and Care. Annals of internal medicine.

28. Kaper JB, Morris JG, Jr., Levine MM (1995) Cholera. Clin Microbiol Rev 8: 48-86.

29. Andrews JR, Basu S (2011) Transmission dynamics and control of cholera in Haiti: an epidemic model. Lancet 377: 1248-1255.
30. Farmer P, Almazor CP, Bahnsen ET, Barry D, Bazile J, et al. (2011) Meeting cholera's challenge to Haiti and the world: a joint statement on cholera prevention and care. PLoS Negl Trop Dis 5: e1145.
31. Bauernfeind A, Fesselet JF, van Herp M, Le Saoût E, Mc Cluskey J, et al. (2004) Cholera Guidelines. In: Frontières MS, editor.
32. Burnham KP, Anderson DR (2002) Model Selection and Multi-Model Inference: A Practical Information-Theoretic Approach.: Springer.
33. Akaike H (1974) A new look at the statistical model identification. IEEE Transactions on Automatic Control 19: 716–723.
34. Gore SM, Fontaine O, Pierce NF (1992) Impact of rice based oral rehydration solution on stool output and duration of diarrhoea: meta-analysis of 13 clinical trials. BMJ 304: 287-291.
35. Molla AM, Ahmed SM, Greenough WB, 3rd (1985) Rice-based oral rehydration solution decreases the stool volume in acute diarrhoea. Bull World Health Organ 63: 751-756.
36. Guarino A, Albano F, Guandalini S (2001) Oral rehydration: toward a real solution. J Pediatr Gastroenterol Nutr 33 Suppl 2: S2-12.
37. Dutta D, Bhattacharya MK, Deb AK, Sarkar D, Chatterjee A, et al. (2000) Evaluation of oral hypo-osmolar glucose-based and rice-based oral rehydration solutions in the treatment of cholera in children. Acta Paediatr 89: 787-790.
38. Yildiz FH, Schoolnik GK (1998) Role of *rpoS* in stress survival and virulence of *Vibrio cholerae*. J Bacteriol 180: 773-784.
39. Heidelberg JF, Eisen JA, Nelson WC, Clayton RA, Gwinn ML, et al. (2000) DNA sequence of both chromosomes of the cholera pathogen *Vibrio cholerae*. Nature 406: 477-483.
40. Kabir S (1982) Characterization of the lipopolysaccharide from *Vibrio cholerae* 395 (Ogawa). Infect Immun 38: 1263-1272.
41. Meibom KL, Blokesch M, Dolganov NA, Wu C-Y, Schoolnik GK (2005) Chitin induces natural competence in *Vibrio cholerae*. Science 310: 1824-1827.
42. De Souza Silva O, Blokesch M (2010) Genetic manipulation of *Vibrio cholerae* by combining natural transformation with FLP recombination. Plasmid 64: 186-195.
43. Borgeaud S, Blokesch M (2013) Overexpression of the *tcp* gene cluster using the T7 RNA polymerase/promoter system and natural transformation-mediated genetic engineering of *Vibrio cholerae*. PLoS One 8: e53952.

44. Bertuzzo E, Azaele S, Maritan A, Gatto M, Rodriguez-Iturbe I, et al. (2008) On the space-time evolution of a cholera epidemic. Water Resour Res 44: W01424.

45. Mari L, Bertuzzo E, Righetto L, Casagrandi R, Gatto M, et al. (2012) Modelling cholera epidemics: the role of waterways, human mobility and sanitation. J R Soc Interface 9: 376-388.

46. Tuite AR, Tien J, Eisenberg M, Earn DJ, Ma J, et al. (2011) Cholera Epidemic in Haiti, 2010: Using a Transmission Model to Explain Spatial Spread of Disease and Identify Optimal Control Interventions. Ann Intern Med 154: 593-601.

47. Koelle K, Rodo X, Pascual M, Yunus M, Mostafa G (2005) Refractory periods and climate forcing in cholera dynamics. Nature 436: 696-700.
